# Photoswitchable precision glycooligomers and their lectin binding

**DOI:** 10.3762/bjoc.10.166

**Published:** 2014-07-15

**Authors:** Daniela Ponader, Sinaida Igde, Marko Wehle, Katharina Märker, Mark Santer, David Bléger, Laura Hartmann

**Affiliations:** 1 Max Planck Institute of Colloids and Interfaces, Department of Biomolecular Systems, Research Campus Golm, 14424 Potsdam, Germany; 2 Max Planck Institute of Colloids and Interfaces, Department of Theory & Bio-Systems, Research Campus Golm, 14424 Potsdam, Germany; 3 Humboldt University, Department of Chemistry, Brook-Taylor-Str. 2, 12489 Berlin, Germany

**Keywords:** azobenzene, glycopolymer, lectin binding, multivalency, multivalent glycosystems, photoswitch, precision polymer

## Abstract

The synthesis of photoswitchable glycooligomers is presented by applying solid-phase polymer synthesis and functional building blocks. The obtained glycoligands are monodisperse and present azobenzene moieties as well as sugar ligands at defined positions within the oligomeric backbone and side chains, respectively. We show that the combination of molecular precision together with the photoswitchable properties of the azobenzene unit allows for the photosensitive control of glycoligand binding to protein receptors. These stimuli-sensitive glycoligands promote the understanding of multivalent binding and will be further developed as novel biosensors.

## Introduction

Carbohydrate ligand–receptor interactions underpin many important processes in biology, for example in host-pathogen interactions [1–2]. Although monosaccharides usually exhibit only low binding affinities, nature is able to obtain high affinity carbohydrate ligands by displaying several monosaccharides/oligosaccharides on a protein scaffold or through a patch of lipids. This is known as the glycocluster effect or the multivalent presentation of sugar ligands [3–4]. This strategy can also be employed for the synthesis of carbohydrate mimetics, where several sugar ligands are attached to a non-natural scaffold. Glycopolymers where natural sugar ligands are presented along a synthetic polymer chain are an emerging class of carbohydrate mimetics [5]. Such glycopolymers offer great potential for various biotechnological and biomedical applications, for antiviral and antibacterial treatments [6]. However, most of these systems are optimized empirically and very little is known about the underlying structure–property relations of glycopolymers. Due to their inherent polydisperse nature and the limitation in controlling precise positioning of functionalities along the backbone, polymer scaffolds make it particularly difficult to correlate their chemical structure with the resulting binding properties.

Recently, we introduced a novel synthetic approach towards monodisperse, sequence-defined glycooligomers, so-called precision glycomacromolecules, via the combination of solid phase polymer synthesis and tailor-made building blocks [7–9]. Through a stepwise assembly of our functional building blocks, we can now control the kind, number, and spacing of sugar ligands along a monodisperse scaffold. Thus, our precision glycomacromolecules allow for direct structure–property correlations and a deeper insight into the multivalent binding of glycomimetics. Through this knowledge we can predict the resulting affinity of a glycomacromolecule based on the number and spacing of sugar ligands attached to the scaffold [7,10–11]. Furthermore, it would be highly interesting to also modulate the binding affinity of a single molecule through a structural change as a response to an external stimulus, for example light.

In order to gain such control over the binding affinity of glycooligomers towards specific lectins, a few studies have recently been dedicated to the construction of photoactive glycoligand incorporating a light-sensitive unit [12–15]. The possibility to photomodulate the complexation of a ligand could lead to a deeper understanding of the typically multivalent binding processes of carbohydrates to proteins, in addition to offer potential perspectives for the sensing and adhesion of bacteriological targets on various substrates. The examples reported so far make use of azobenzene [16], a well-known photochromic compound offering robustness and straightforward preparation. It is able to reversibly isomerize between an extended and planar form (*E*-isomer, thermodynamically favored) and a more compact and twisted state (*Z*-isomer). Monovalent [15] and divalent [16] photoswitchable glycoconjugates described in the literature showed a different binding behavior depending on the configuration of the azobenzene (*E* or *Z*), although the effect was rather modest.

We anticipated that the photomodulation of the binding activity could be enhanced within more complex architectures, i.e., divalent and trivalent glycooligomers incorporating one or two photoswicthes in the backbone, as a result of a large photoinduced geometrical change in the oligomer shape and concomitantly in the sugar ligands accessibility. Indeed, dramatic shrinking of rigid-rod polymers for example, occurs upon photoirradiation when several azobenzenes are introduced in the main-chain, the embedded photoswitches acting as hinges [17–18].

**Figure 1:**
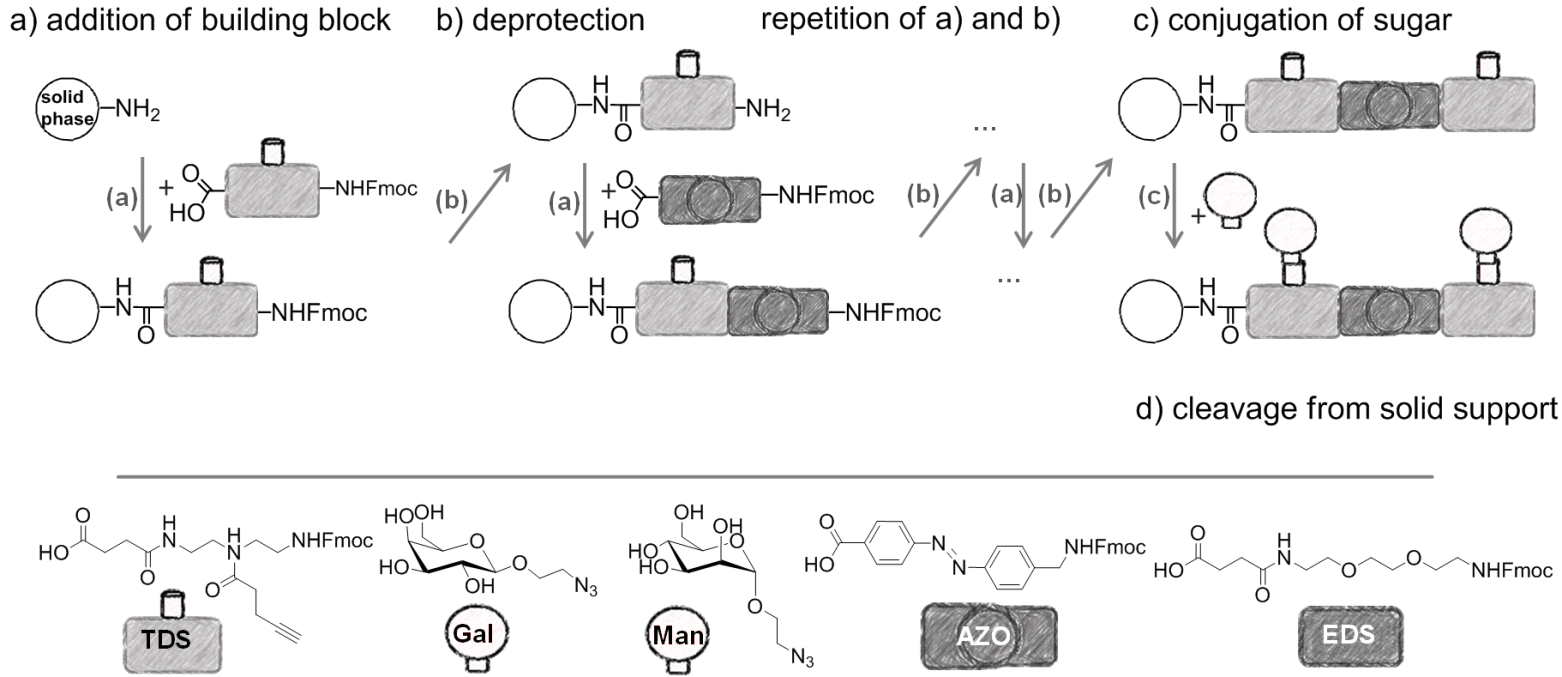
Synthesis of photoswitchable precision glycooligomers via stepwise addition of building blocks on solid support followed by on-resin functionalization of alkyne side chains with sugar azide ligands and final cleavage from the support.

In the present study, an azobenzene functionalized with an Fmoc-protected aminomethyl group and a carboxylic acid both *para* to the N=N bond was used as one building block during solid-phase polymer synthesis of precision glycomacromolecules (see AZO, Figure 1) [19–22]. The benzylamine fragment was favored over the phenylamine one for two reasons: first, its higher nucleophilicity allows for a smoother synthesis and second, the resulting *Z*-azobenzene exhibits a high thermal stability as compared to the fully conjugated push-pull azobenzene based on the phenylamine fragment. In total, five precision glycooligomers were synthesized containing up to two azobenzene units in the oligomeric backbone and presenting galactose residues in the side chains. As control structures, glycooligomers containing either a hydrophilic flexible linker unit instead of the azobenzene moiety, or mannose instead of galactose ligands were synthesized. The photoswitchable behavior of all azobenzene-containing glycooligomers was evaluated along with their photoswitchable binding affinities towards PA-IL (also called LecA) as targeted lectin receptor [23].

## Results and Discussion

### Synthesis of photoswitchable precision glycooligomers

The synthesis of photoswitchable precision glycooligomers is based on the previously developed solid-phase assembly of functional dimer building blocks [7,24–25]. The term dimer building block refers to the coupling of diacid and diamine building blocks in solution prior to solid phase coupling. The obtained building blocks contain a free carboxy- and an Fmoc-protected amino group and thus can be coupled via standard peptide coupling protocols giving a polyamide backbone. Additionally, dimer building blocks can carry functional groups as side groups or in the main chain, thereby allowing for the conjugation of sugar ligands and the control of the backbone properties. Three different dimer building blocks were employed for the synthesis of the photoswitchable glycooligomers: the triple bond-functionalized building block TDS [7], an ethylene glycol spacer building block EDS [7], and the photoswitchable building block AZO. TDS allows for the conjugation of sugar azide ligands via the copper-catalyzed azide–alkyne cycloaddition (CuAAC). The synthesis and coupling on solid support of TDS and EDS has been previously described [7,10]. AZO was synthesized via Mills coupling adapting literature protocols [20–22].

Five glycooligomers were synthesized – three photoswitchable glycooligomers containing the AZO building-block and two non-switchable control structures containing EDS instead of AZO (Table 1). The synthesis proceeded following standard peptide coupling protocols followed by introduction of the sugar ligands (Figure 1): Starting from an ethylenediamine functionalized trityl resin (0.0125 mmol), the first building block, i.e., TDS (8 equiv) was attached via activation with PyBOP/HOBt (8 equiv/4 equiv) and DIPEA (16 equiv) in DMF and subsequent coupling for 1 hour. After a washing step, the terminal Fmoc protecting group was cleaved by treatment with 25% piperidine in DMF three times for 5, 10 and 15 minutes. After complete removal of the Fmoc protecting group, the second building block (AZO or EDS) was coupled following the same reaction conditions. After repetition of the coupling/deprotection steps, the oligomeric backbone was formed on the solid support. In the next step, the sugar ligands were introduced to the oligomeric backbone via CuAAC. To this end, two sugar azides (2-azidoethyl galactoside and 2-azidoethyl mannoside) were previously synthesized following literature protocols [26]. 8 equiv of sugar azide, 20 mol % sodium ascorbate and 20 mol % CuSO_4_ per alkyne group were dissolved in DMF/H_2_O, added to the resin and shaken for 4 hours. Excess reagents as well as the copper catalyst were removed by washing with a 23 mM solution of sodium diethyl dithiocarbamate in DMF as well as water and DCM. The final precision glycooligomers were obtained after cleavage from the resin using 30 vol % TFA in DCM and isolated after precipitation from cold Et_2_O. Crude products containing AZO building blocks were obtained with ~80% purity as determined by RP-HPLC (see Supporting Information File 1) and further purified by preparative RP-HPLC. Glycooligomers containing EDS building blocks were obtained in high purity (>95%) directly after cleavage. After purification, all final products were obtained in ~60% yield and >90% purity as confirmed by ESIMS, HPLC and NMR (see Supporting Information File 1).

**Table 1:** List of photoswitchable precision glycooligomers obtained via solid phase polymer synthesis.

Entry	Sample name	Chemical structure

1	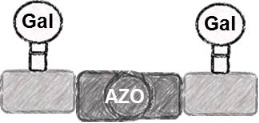 **Azo-Gal(1,3)-3**	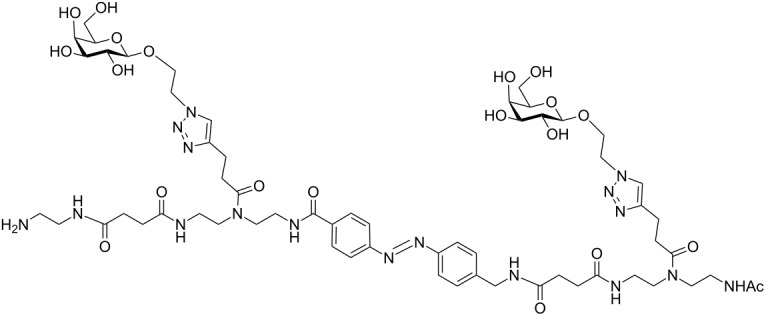
2	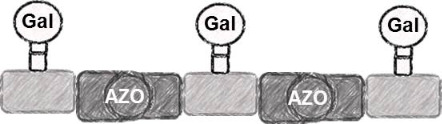 **Azo-Gal(1,3,5)-5**	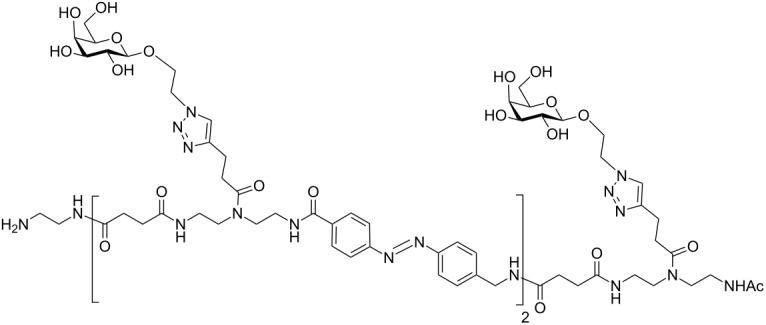
3	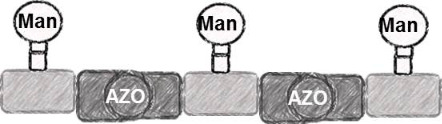 **Azo-Man(1,3,5)-5**	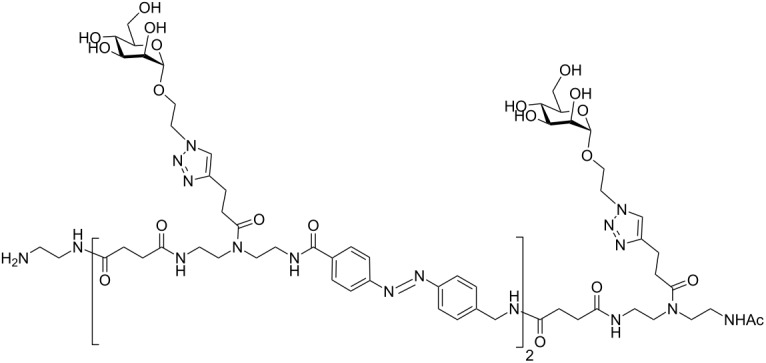
4	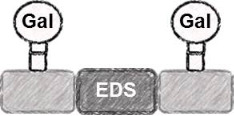 **EDS-Gal(1,3)-3**	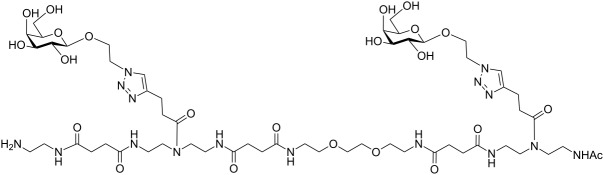
5	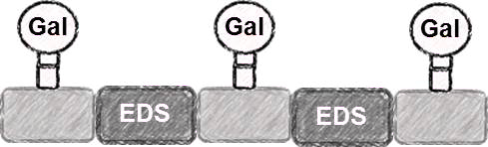 **EDS-Gal(1,3,5)-5**	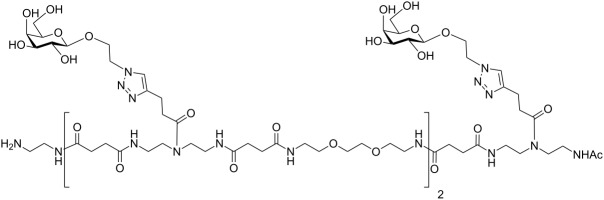

### Characterization of the photoswitchable properties

**Figure 2:**
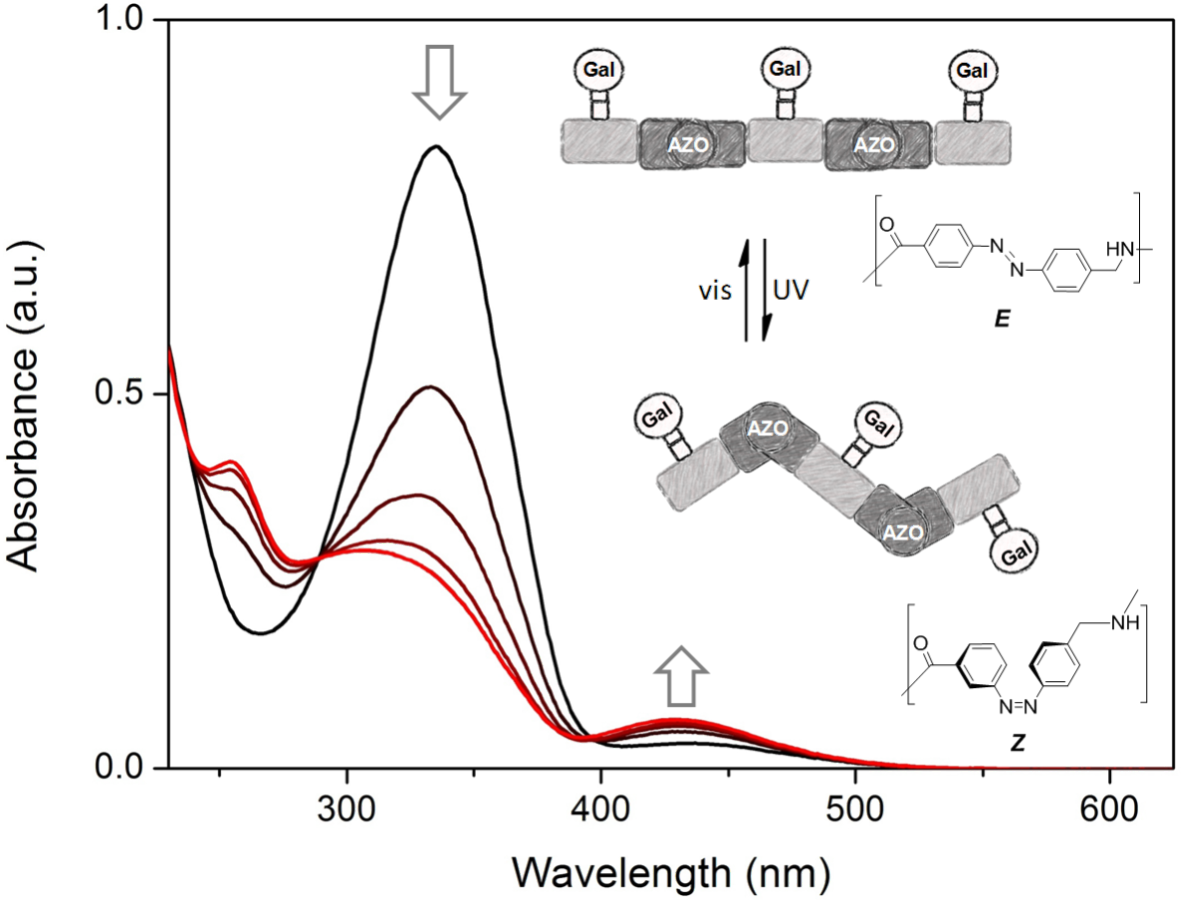
Characterization of the *E* → *Z* photoisomerization (λ = 360 nm) of **Azo-Gal(1,3,5)-5** in buffer solution at 25 °C via UV–vis absorption spectroscopy measurements.

The photochromic behavior of the newly synthesized AZO-glycooligomers was investigated by UV–vis absorption spectroscopy and ultraperformance liquid chromatography (UPLC). Aqueous solutions of **Azo-Gal(1,3)-3** and **Azo-Gal(1,3,5)-5** (the compounds were dissolved in the buffer used for the SPR measurements, see Supporting Information File 1 for the details) were irradiated at λ = 360 nm to induce the *E* → *Z* isomerization. The typical decrease of the π→π* band at 330 nm and increase of the n→π* band centered at 440 nm was observed in the UV–vis absorbance spectra of both photoswitchable glycooligomers (see Figure 2 for the spectrum of **Azo-Gal(1,3,5)-5**). The presence of well-defined isosbestic points at 290 nm and 395 nm indicates a clean photoisomerization process. The composition at the photostationary state (PSS) was analyzed by UPLC using integration of the UV signal at the wavelengths of the isosbestic points. Starting from an **Azo-Gal(1,3,5)-5** aqueous solution containing mainly the *E,E*-isomer, irradiation in the UV-region led to a majority of *Z,Z*-isomer (70%), together with significant quantities of mixed isomers (10% of *E*,*Z* and 10% of *Z*,*E*), whereas 10% of *E,E*-isomer did not isomerize (see Supporting Information File 1 for more details). This values correspond to a total amount of 80% of *Z*-azobenzenes in the PSS mixture, in accordance to the 78% of *Z*-azobenzene found in the PSS solution of **Azo-Gal(1,3)-3** upon irradiation at 360 nm, indicating that the two photochromic units of **Azo-Gal(1,3,5)-5** operate independently. The *Z* → *E* back-isomerization was triggered upon illumination at λ > 400 nm, leading to solutions containing 92% of *E*-isomers. Further *Z*/*E* isomerization cycles could be performed similarly without affecting the PSS ratio, demonstrating the reversibility of the systems. Finally, the thermal stability of *Z*-**Azo-Gal(1,3)-3** in the buffer solution is quite high. Indeed, after 48 h at 25 °C only 12% of the *Z*-isomers converted back to the *E*-isomer (see Supporting Information File 1). The thermal stability of the trivalent **Azo-Gal(1,3,5)-5 ***Z-*isomers is anticipated to be very similar since their two azo units were found to operate independently. The long-lived *Z-*forms of these glycoconjugates allows for a convenient handling of the PSS mixtures and a precise measurement of their binding affinities.

### Lectin binding studies

#### Competition binding assay

After the photophysical characterization of the new glycooligomers containing photoswitchable azobenzene moieties in the backbone, their ability to bind to a protein receptor via their sugar ligands was investigated. Depending on the properties of the backbone (EDS vs AZO moieties and *E-* vs *Z*-configuration), we can expect differences in the glycooligomer-receptor binding mode and thus in the resulting glycooligomer ligand affinity.

**Figure 3:**
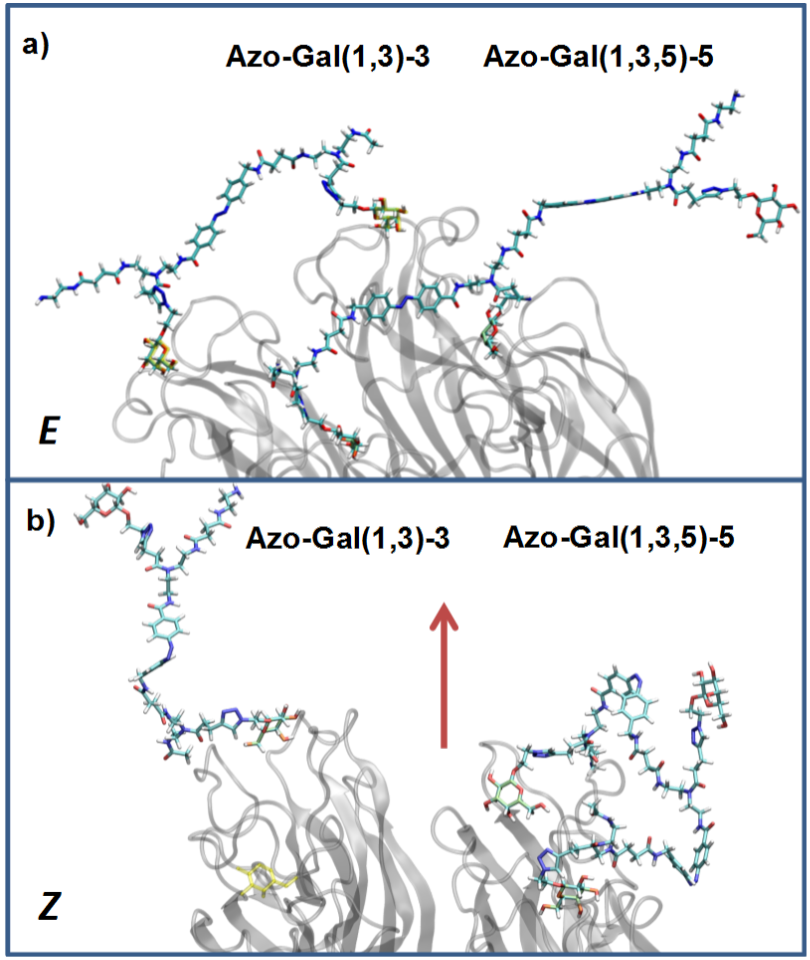
Structural models of **Azo-Gal(1,3)-3** and **Azo-Gal(1,3,5)-5** in (a) *E*- and (b) all-*Z*-configurations of the connecting azobenzene groups. The dimer is shown to the left, the trimer to the right. To facilitate the representation in (b), the whole complex has been rotated around the indicated in-plane axis (arrow). The PA-IL protein structure has been inferred from the Protein Data Bank (PDB code 4ljh).

As targeted receptor, we chose PA-IL, a tetrameric, calcium dependent lectin specifically binding to α-galactoside and β-galactoside structures [27]. It is composed of 121 amino acids (51 kDa) associated as homotetramers [28]. The crystal structure reveals a tetrameric arrangement with a general rectangular shape with the smaller distance of binding sites being 2.6 nm and the longer one being 7.9 nm [27]. Thus, in the *E*-configuration, the distance between two neighboring sugar ligands on the di- and trivalent glycooligomers can span the distance of two neighboring binding sites and potentially allow for chelate binding (see Figure 3). Upon switching to the *Z*-configuration this distance will decrease and therefore can be expected to strongly impact the ligand–receptor binding and thus the resulting binding affinity.

In order to determine the binding affinity of the precision glycooligomers to PA-IL, surface plasmon resonance (SPR) experiments were performed. At first, an inhibition/competition assay was carried out. The SPR chip was modified with a β-D-galactose-polymer and α-D-mannose-polymer as negative control (see Supporting Information File 1). In a first experiment, binding of PA-IL (1 µM) to the chip was determined and set as 100%. In a second set of experiments, binding of preincubated mixtures of PA-IL (1 µM) and glycooligomers at serial dilutions (400 µM to 0.1 µM) were measured. Binding of glycooligomer to PA-IL resulted in a decrease of PA-IL binding on the chip. Plotting this concentration dependent decrease in binding against time, sigmoidal curves were obtained and fitted with the Hill equation. The inhibitory concentration at 50% binding (IC_50_) was derived for the different glycooligomers (Table 2).

**Table 2:** IC_50_ values obtained by SPR inhibition/competition assays of the photoswitchable glycooligomers and control structures.

#	Compound name		IC_50_ [µM]

1	**AZO-Gal(1,3)-3**	*E*	5.7 ± 1.7
		PSS^a^	9.4 ± 0.1
2	**AZO-Gal(1,3,5)-5**	*E*	3.4 ± 0.4
		PSS^a^	4.1 ± 1.3
3	**AZO-Man(1,3,5)-5**		n.b.^b^
4	**EDS-Gal(1,3)-3**		3.2 ± 0.2
5	**EDS-Gal(1,3,5)-5**		2.0 ± 0.6
6	**β-Me-Gal**		55 ± 6

^a^PSS: photostationary state @ 360 nm; ^b^n.b.: no binding.

All galactose-containing glycooligomers can bind to PA-IL. The mannose-containing control oligomer does not show any binding. This confirms that the backbone itself does not undergo non-specific interactions with the receptor, as we could previously also show for glycooligomers binding to Concanavalin A (Con A) lectin receptor [7,10–11]. All multivalent glycooligomers show a decrease in IC_50_, i.e., an increase in binding affinity in comparison to the monovalent β-methyl galactoside. Overall, IC_50_ values are in the µM range, with the EDS based di- and trivalent glycooligomers giving the highest binding affinity with IC_50_ values of 2.0 and 3.2 µM, respectively. The corresponding di- and trivalent oligomers containing the AZO spacer instead of the EDS unit show higher IC_50_ values for both *E*- and *Z*-isomers, i.e., a slightly less favorable binding to the receptor.

Comparing the binding behavior of the *E*-glycooligomers vs their corresponding PSS mixtures, we see that the IC_50_ values for the divalent glycooligomer **Azo-Gal(1,3)-3** shows a significant decrease in binding affinity upon switching (entry 1 Table 2), whereas the trivalent glycooligomer **Azo-Gal(1,3,5)-5** binds with the same affinity before and after switching (entry 2 Table 2). In order to gain a first insight into the possible conformations of the glycooligomers and thus their potential binding modes, we performed molecular modeling as outlined in the caption of Figure 3. This data suggests that the trivalent **Azo-Gal(1,3,5)-5** can bind with two sugar ligands to the PA-IL receptor in both *E* and *Z*-configurations. In the all-*E* configuration two neighboring sugar ligands can span the distance of two neighboring binding sites (Figure 3a). Through the change in conformation for the all-*Z* configuration, the overall distance between the sugar ligands decreases, now presenting the two terminal sugar ligands with the same distance as the neighboring sugar ligands in the *E*-configuration (Figure 3b), and thus again allowing for a bivalent binding to the receptor. This model is supported by the experimental finding that there is no change in binding affinity from the *E*- to the *Z*-glycooligomer.

In contrast to this, the divalent glycooligomer (**Azo-Gal(1,3)-3**) shows a significant decrease in binding affinity from an IC_50_ value of 5.7 ± 1.7 µM for the *E*-form to 9.4 ± 0.1 µM in the PSS. This indicates a change in the accessibility of the sugar ligands for receptor binding, with the *Z*-oligomer having a less accessible conformation. Following the same model as for the trivalent ligand, the divalent ligand has the opportunity to bind in a bivalent fashion in its *E*-form (Figure 3a) while the *Z*-form only allows for a monovalent binding of one of the sugars to the protein receptor (Figure 3b). Such a change in binding mode is expected to lead to the observed decrease in binding affinity. It is important to note that additional binding modes might contribute to the multivalent binding of the glycooligomer ligands as well. Further studies will evaluate in more detail different potential binding modes such as chelate binding, intermolecular crosslinking, and rebinding effects.

In order to approach practical applications of a photoswitchable device that could modulate its binding affinity towards PA-IL lectins on demand, we were intrigued in the ability of our light-responsive systems to work when immobilized on a surface. To this end we attached the photoswitchable precision glycooligomers directly to the SPR chip and performed a second set of lectin binding experiments using SPR and measuring PA-IL binding on the chip. The glycooligomers were covalently linked to the chip via their terminal amine group and the simultaneous activation of the carboxyl-functionalized chip surface as *N*-hydroxysuccinimide (NHS) ester (see Supporting Information File 1). Successful functionalization was monitored by SPR. Photoswitching of the glycooligomers was realized either ex situ prior to surface functionalization or in situ by direct irradiation of the functionalized chip. The equilibrium constant *K*_D_ was obtained by fitting the obtained binding values at the turning point between binding- and dissociation-curve with a steady-state affinity model (Biacore T100 Evaluation Software 2.0.3). In agreement with the previously determined IC_50_ values, all *K*_D_ values are in the µM range.

We observed a significant difference for the ex situ irradiated samples in comparison to irradiation of the glycooligomers directly on the chip. The *K*_D_ values showed no difference in binding affinity between *E*- and *Z*-isomers when the chip was irradiated directly. This indicates that either the light could not penetrate efficiently through the organic layer of the chip, or more likely that photoswitching was prohibited due to a lack of conformational freedom, as often observed in the solid state. However, for the ex situ irradiated glycooligomers, we observed a significant decrease in binding affinity for **Azo-Gal(1,3,5)-5** from *K*_D_ = 3.3 ± 0.3 for the *E*-form to *K*_D_ = 7.4 ± 0.9 in the PSS, whereas **Azo-Gal-(1,3)-3** shows a small difference in lectin binding before and after photoswitching (entry 1, Table 3). This is in contrast to the previous finding of the inhibition/competition assay, where after photoswitching the **Azo-Gal(1,3,5)-5** was unaffected while **Azo-Gal-(1,3)-3** showed a significant difference in binding.

**Table 3:** *K*_D_ values obtained by SPR direct binding assay of the photoswitchable glycooligomers (PSS: photostationary state @ 360 nm).

**Azo-Gal(1,3)-3**	*K*_D_ [µM]	**Azo-Gal(1,3,5)-5**	*K*_D_ [µM]

*E*	1.7 ± 0.1	*E*	3.3 ± 0.3
PSS (ex situ irradiation)	2.4 ± 0.1	PSS (ex situ irradiation)	7.4 ± 0.9
PSS (irradiation on chip)	1.8 ± 0.1	PSS (irradiation on chip)	3.2 ± 0.9

In contrast to the inhibition/competition assay where both components were in solution, now the glycooligomer is attached via one chain end to the chip surface. Thus, the degree of functionalization of the surface as well as the surface-oligomer linker has to be taken into consideration. From the values of refractive index determined via SPR on the glycooligomer functionalized chip, we can assume a similar degree of surface functionalization for all switched and non-switched glycooligomers (see Supporting Information File 1). Since all glycooligomers were attached directly via their free *N*-terminus (see Table 1) without any additional linker moieties, we believe that the first sugar ligand, i.e., the closest to the NHS-connection, might not be accessible for interactions with the receptor. Thus the divalent **Azo-Gal(1,3)-3** is reduced to an effective monovalent ligand. Independent of their *E*- or *Z*-configurations, the divalent glycooligomers can only bind in a monovalent fashion explaining that no difference in binding affinity was observed upon photoswitching. Following this hypothesis, trivalent **Azo-Gal(1,3,5)-5** would be reduced to an effective divalent ligand upon attachment to the SPR chip. Therefore **Azo-Gal(1,3,5)-5** should now show similar changes in binding behavior as were previously measured for divalent **Azo-Gal(1,3)-3** in solution**.** Indeed, we observe a similar decrease in binding affinity from *K*_D_ = 3.3 ± 0.3 for the *E*-form to *K*_D_ = 7.4 ± 0.9 in the PSS.

## Conclusion

We have presented the straightforward synthesis of a series of photoswitchable glycooligomers by combination of solid phase polymer synthesis and functional building blocks. We could show that the azobenzene moieties introduced into the oligomeric backbone retain their photoswitchable behavior and thus allow for a light-induced change in the geometry of the glycooligomers. Binding studies of the galactose-functionalized glycooligomers showed specific binding to PA-IL and a controlled reduction in binding affinity upon *E* → *Z* photoisomerization. We proposed a first model to explain our findings based on molecular modelling for ligand binding. Ongoing studies further investigate ligand binding by additional techniques such as isothermal titration calorimetry and fluorescence spectroscopy. Overall, we have successfully developed photoswitchable glycomimetics that allow for a stimulus-induced change in binding affinity. Therefore we will further explore our photoswitchable glycooligomers as tunable glycomimetic ligands and their potential for a variety of biotechnological and biomedical applications such as the sensing and isolation of bacteria as well as the development of antibacterial treatments.

## Experimental

AZO building block synthesis: *N*-Fmoc-*para*-(aminomethyl)phenylazobenzoic acid was prepared adapting literature procedures [19,23] (see Supporting Information File 1).

### General solid phase coupling protocols

**General coupling protocol:** Commercially available trityl-tentagel-OH resin was modified with an ethylenediamine linker and used as resin for solid phase synthesis. 0.0125 mmol of resin were swollen in DCM for 15 min. The initial coupling to the ethylenediamine linker was performed with a 0.1 mmol building block solution (8 equiv, TDS, EDS or AZO) in DMF (0.5 mL), followed by the addition of a 0.1 mmol PyBOP solution (8 equiv) together with 0.05 mmol HOBt (4 equiv) and 0.2 mmol (16 equiv) DIPEA in DMF (0.1 mL). This solution was added to the resin. After shaking for one hour the resin was washed from excessive reagent with DMF.

**General CuAAC protocol:** 0.1 mmol (8 equiv) of 2-azidoethyl pyranoside per alkyne group, dissolved in 1 mL DMF was added to 0.0125 mmol of resin loaded with EDS/AZO and TDS building blocks. 20 mol % sodium ascorbate per alkyne group and 20 mol % CuSO_4_ per alkyne group were dissolved in 0.5 mL of water and also added to the resin. The resulting mixture was shaken for at least four hours. After that, the resin was washed with a 23 mM solution of sodium diethyl dithiocarbamate in DMF, water, DMF and DCM.

**Fmoc cleavage:** The Fmoc protecting group was cleaved by the addition of a solution of 25% piperidine in DMF three times for 5, 10 and 15 minutes, respectively. This was followed by carefully washing the resin with DMF.

**Capping of N-teminal site:** The free primary amine, obtained after final Fmoc cleavage, was capped with an acetyl group by the addition of 2.5 mL acetic anhydride. After shaking the mixture for 15 min, the resin was washed with DMF and DCM.

**Cleavage from solid phase:** 30% TFA in DCM was added to the resin and the mixture was shaken for one hour. The filtrate was added to cold diethyl ether (40 mL) resulting in white precipitate. This was centrifuged and the ether decanted. The crude product was dried in N_2_ stream, dissolved in water (1 mL) and lyophilized.

**Azo-Gal(1,3)-3:** This structure was synthesized by applying the general coupling protocol three times with building blocks in the sequence TDS, AZO, TDS. After capping the primary amine, two galactose units were conjugated to the scaffold according to the general CuAAC protocol. The product was cleaved from the resin as final step giving 13 mg (yield: 75%). ^1^H NMR (400 MHz, D_2_O) δ 7.84–7.73 (m, 12H), 7.54–7.48 (m, 2H), 4.50–4.42 (m, 9H), 4.29–4.07 (m, 4H), 4.07 (br. s, 2H), 3.78 (s, 4H), 3.67–2.48 (m, 50 H), 1.85 (d, *J* = 21.0 Hz, 3H) ppm; RP-HPLC (5%/95% MeCN/H_2_O → 30%/70% MeCN/H_2_O in 30 min): *t*_R_ = 14.8 min;. ESIMS [M + H]^+^: calcd for C_60_H_89_N_17_O_20_, 1368.6; found, 1368.4; [M + 2H]^2+^ 684.8; found, 684.8, [M + 3H]^3+^ 456.9; found, 457.0.

**AZO-Gal(1,3,5)-5:** This structure was synthesized by applying the general coupling protocol five times with building blocks in the sequence TDS, AZO, TDS, AZO, TDS. After capping the primary amine, three galactose units were conjugated to the scaffold according to the general CuAAC protocol. The product was cleaved from the resin as final step giving 18 mg (yield: 68%). ^1^H NMR (400 MHz, D_2_O) δ 7.73–7.45 (m, 12H), 7.34 (br. s, 3H), 4.48–4.31 (m, 9H), 4.25–4.08 (m, 8H), 3.78–3.66 (m, 8H), 3.66–3.30 (m, 32H), 3.18 (br. s, 4H), 2.98 (br. s, 3H), 2.98–2.35 (m, 25H), 1.82 (d, *J* = 10.6 Hz, 3H) ppm; RP-HPLC (5%/95% MeCN/H_2_O → 30%/705% MeCN/H_2_O in 30 min): *t*_R_ = 21.7 min; ESIMS [M + 2H]^2+^ calcd for C_95_H_134_N_26_O_30_, 1060.5; found, 1060.4; [M + H + Na]^2+^ 1082.9; found, 1082.6; [M + 3H]^3+^ 707.4; found, 707.5, [M + 4H]^4+^ 530.7; found, 530.8.

**AZO-Man(1,3,5)-5:** This structure was synthesized by applying the general coupling protocol five times with building blocks in the sequence TDS, AZO, TDS, AZO, TDS. After capping the primary amine, three mannose units were conjugated to the scaffold according to the general CuAAC protocol. The product was cleaved from the resin as final step giving 17 mg (yield: 64%). ^1^H NMR (400 MHz, D_2_O) δ 7.68–7.48 (m, 12H), 7.32 (br. s, 3H), 4.69–4.58 (m, 3H), 4.41–4.30 (m, 8H), 3.90 (br. s, 2 H), 3.75–3.70 (m, 6H), 3.62–3.08 (m, 48H), 2.86–2.45 (m, 32H), 1.82 (d, *J* = 8.5 Hz, 3H) ppm; RP-HPLC (5%/95% MeCN/H_2_O → 30%/705% MeCN/H_2_O in 30 min): *t*_R_ = 21.7 min; ESIMS [M + H + Na]^2+^calcd for C_95_H_134_N_26_O_30_, 1082.9; found, 1082.8; [M + 3H]^3+^ 707.4; found, 707.5; [M + 4H]^4+^ 530.7; found, 530.8.

**EDS-Gal(1,3)-3:** This structure was synthesized by applying the general coupling protocol three times with building blocks in the sequence TDS, EDS, TDS. After capping the primary amine, two galactose units were conjugated to the scaffold according to the general CuAAC protocol. The product was cleaved from the resin as final step giving 21 mg (yield: quant). ^1^H NMR (400 MHz, D_2_O) δ 8.00 (s, 2H), 4.44 (d, *J* = 7.8 Hz, 2H), 4.40–4.32 (m, 2H), 4.21–4.12 (m, 2H), 3.99–3.96 (m, 2H), 3.83–3.79 (m, 4H), 3.78–3.64 (m, 16H), 3.59–3.50 (m, 12H), 3.43 (t, *J* = 10.1 Hz, 12H), 3.21 (t, *J* = 5.8 Hz, 2H), 3.08 (t, *J* = 7.0 Hz, 4H), 2.86 (t, *J* = 6.9 Hz, 4H), 2.62–2.52 (m, 12H), 2.00 (d, *J* = 6.0 Hz, 3H) ppm; RP-HPLC (5%/95% MeCN/H_2_O → 30%/70% MeCN/H_2_O in 60 min): *t*_R_ = 12.9 min; ESIMS [M + 2H]^2+^ calcd for C_56_H_96_N_16_O_23_, 681.3; found, 681.3; [M + 3H]^3+^ 454.6; found, 454.6.

**EDS-Gal(1,3,5)-5** [9]: This structure was synthesized by applying the general coupling protocol five times with building blocks in the sequence TDS, EDS, TDS, EDS, TDS. After capping the primary amine, three galactose units were conjugated to the scaffold according to the general CuAAC protocol. The product was cleaved from the resin. ^1^H NMR (400 MHz, D_2_O) δ 8.04 (d, *J* = 7 Hz, 3H), 4.74 (br. s, 6H), 4.43 (d, *J* = 8 Hz, 3H), 4.38–4.33 (m, 3H), 4.19–4.14 (m, 3H), 3.97 (s, 3H), 3.81–3.79 (m, 7H), 3.72 (s, 10H), 3.65 (m, 10H), 3.56–3.51 (m, 16H), 3.44–3.39 (m, 18H), 3.20 (t, *J* = 6 Hz, 2H), 3.08 (t, *J* = 6 Hz, 6H), 2.85 (t, *J* = 7 Hz, 6H), 2.58–2.50 (m, 20H), 1.99 (d, *J* = 5 Hz, 3H) ppm; RP-HPLC (5%/95% MeCN/H_2_O → 30%/70% MeCN/H_2_O in 60 min) *t*_R_ = 14.1 min; ESIMS [M + 2H]^2^ calcd for C_87_H_148_N_24_O_36_, 1053.5; found, 1053.8, [M + 3H]^3+^ 702.7; found, 702.8, [M + 4H]^4+^ 527.3; found, 527.4, [M + 5H]^5+^ 422.0; found, 422.2.

## Supporting Information

File 1 Further experimental procedures, characterization data and spectra.
