# Supplementary material for: Photoswitchable precision glycooligomers and their lectin binding
Source: Beilstein J Org Chem. 2014 Jul 15;10:1603–12. doi: 10.3762/bjoc.10.166 (PMC4143111; doi:10.3762/bjoc.10.166)
Supplement: File 1 — Further experimental procedures, characterization data and spectra. [file Beilstein_J_Org_Chem-10-1603-s001.pdf]

Supporting Information

for

Photoswitchable precision glycooligomers and their lectin binding

Daniela Ponader[1], Sinaida Igde[1], Marko Wehle[2], Katharina Märker[1], Mark Santer[2], David Bléger[3]* and Laura Hartmann[1]*

Address: [1]Max Planck Institute of Colloids and Interfaces, Department of Biomolecular Systems, Research Campus Golm, 14424 Potsdam, Germany, [2]Max Planck Institute of Colloids and Interfaces, Department of Theory & Bio-Systems, Research Campus Golm, 14424 Potsdam, Germany and [3]Humboldt University, Department of Chemistry, Brook-Taylor-Str. 2, 12489 Berlin, Germany

Email: Laura Hartmann - laura.hartmann@mpikg.mpg.de, David Bléger*- david.bleger@chemie.hu-berlin.de

*Corresponding author

Further experimental procedures, characterization data and spectra

Synthesis of AZO-building block

N-Fmoc-*para*-(aminomethyl)phenylazobenzoic acid was prepared adapting literature procedures [1]. *Para*-nitrosobenzoic acid [2] (0.94 g, 6.2 mmol) and *N*-Fmoc-*para*-aminobenzylamine [3] (1.06 g, 3.1 mmol) were suspended in acetic acid/DMSO 1:1 (80 mL) and stirred at room temperature for 48 h. Water was added to the reaction mixture, the precipitate was filtered, washed with water several times, dried, and recrystallized from acetone/water mixtures yielding the desired azo compound (660 mg, 45%) as an orange solid.

^{1}H-NMR (500 MHz, DMSO-d_6): δ (ppm) = 8.11 (d, *J* = 8.3 Hz, 2H), 7.92 (d, *J* = 8.3 Hz, 2H), 7.85 (d, *J* = 8.3 Hz, 4H), 7.68 (d, *J* = 7.3, 2H), 7.40-7.31 (m, 4H), 7.31 (t, *J* = 7.3 Hz, 2H), 4.37 (d, *J* = 6.6 Hz, 2H), 4.26 (d, J = 5.9 Hz, 2H), 4.21 (t, *J* = 6.4 Hz, 1H). HRMS-ESI: *m/z* = 476.1631 (calcd for $[M-H]^-$, 476.1610).

Photochemical isomerization

UV–visible absorption spectra were recorded using quartz cuvettes on a Cary 50 spectrophotometer equipped with a Peltier-thermostated cell holder (precision of 0.1 °C). Irradiation experiments were performed using a LOT-Oriel 1000 W medium-pressure Xenon lamp equipped with cut-off filters (λ_{max} = 360 nm with FWHM = 43 nm for the *E* → *Z* isomerization, and λ > 400 nm for the *Z* → *E* isomerization).

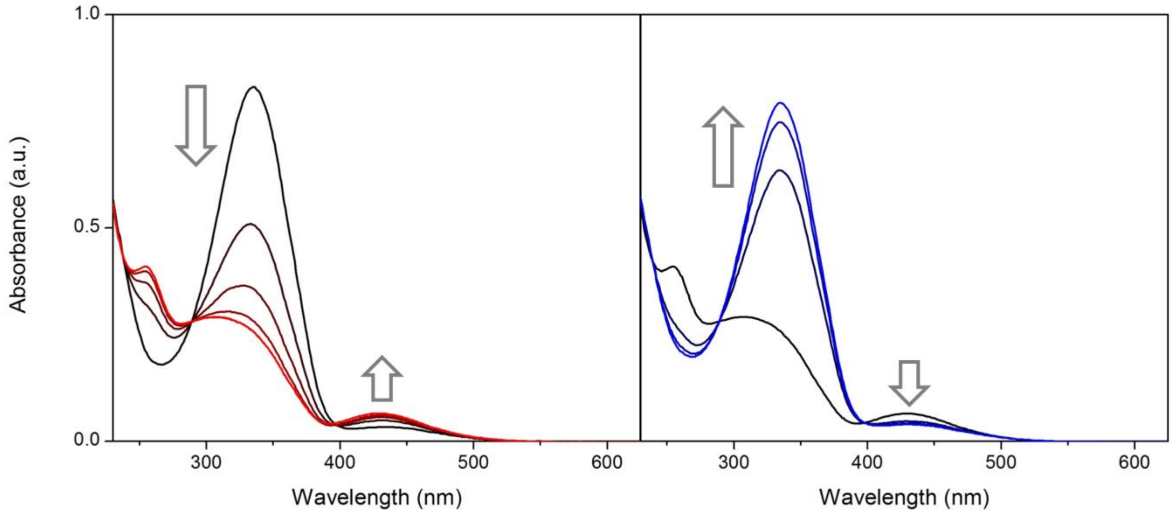

Figure S1: Time-evolution UV–vis absorption spectra of *E*-**Azo-Gal(1,3,5)-5** in buffer solution upon irradiation at λ = 360 nm until the PSS is reached (left), and *Z* → *E* reverse isomerization photoinduced at λ > 400 nm within the same sample (right).

UPLC analysis of the PSSs: Ultra performance liquid chromatography coupled to mass spectrometry detection was performed with a Waters Alliance systems (gradient mixtures of acetonitrile/water) equipped with Acquity UPLC columns. The Waters systems consisted of a Waters Separations Module 2695, a Waters Diode Array detector 996, a LCT Premier XE mass spectrometer, and a Waters Mass Detector ZQ 2000.

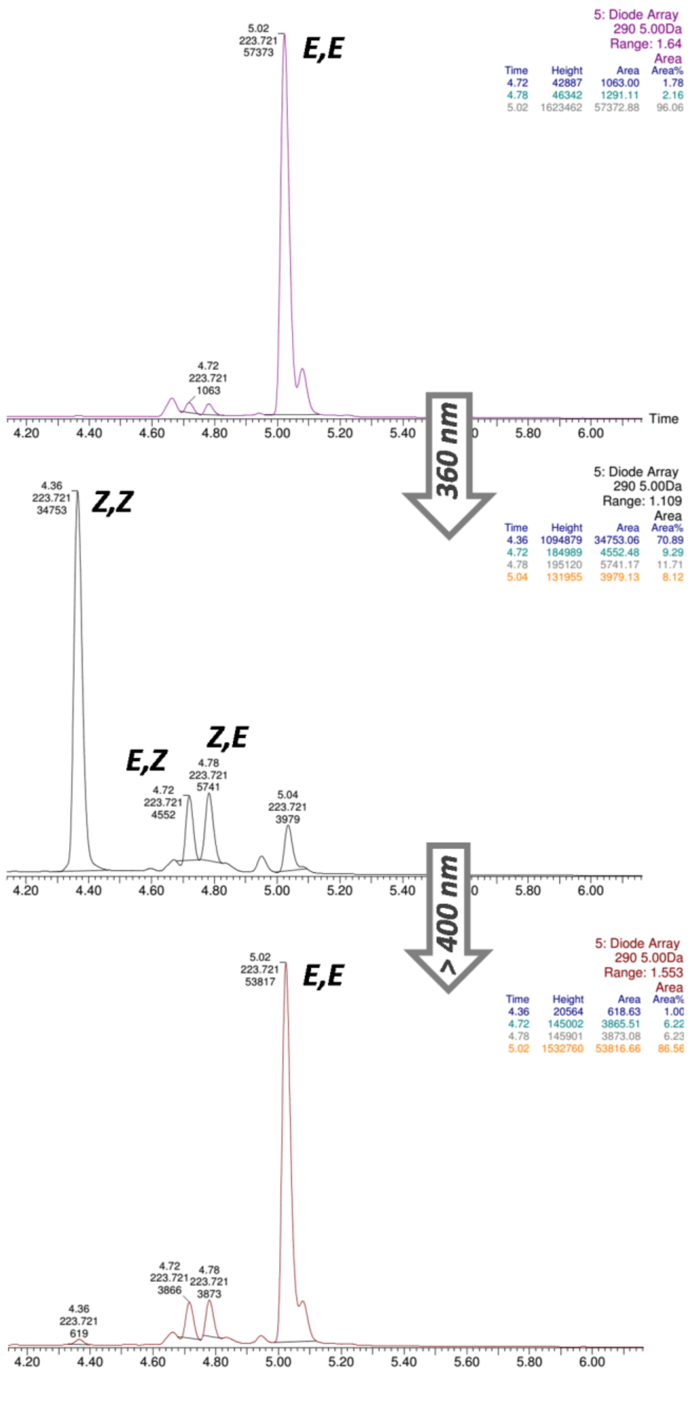

FigureS2: UPLC-traces (recorded at λ(*isosbestic point*) = 290 nm) of **Azo-Gal(1,3,5)-5** in buffer solution before irradiation (top), after irradiation at λ = 360 nm (middle), and λ > 400 nm (bottom).

Thermal *Z* → *E* isomerization:

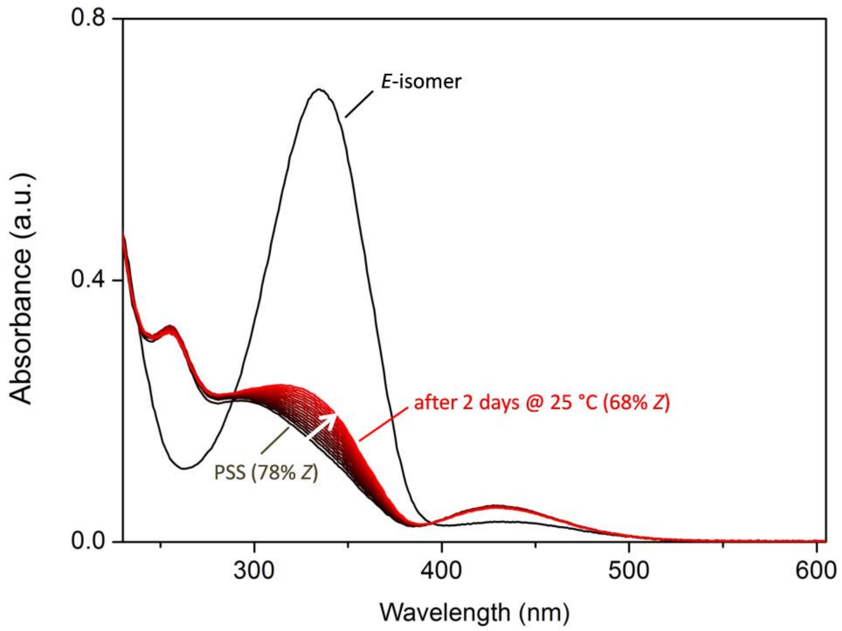

Figure S3: UV–vis absorption spectra of *E*-**Azo-Gal(1,3)-3** in buffer solution, its PSS mixture upon irradiation at 360 nm, and the time-evolution spectra of the PSS mixture at 25 °C over 45 hours (the curves were recorded with 3 h intervals).

SPR PA-IL inhibition/competition studies:

A sensor chip SA coated with streptavidin was conditioned with three consecutive injections of 100 µl 1 M NaCl and 50 mM NaOH in a flow rate of 100 µL/min. Then, biotinylated β-D-galactose-PAA (0.1 nM in HBS-EP buffer) was immobilized on flow cell 2 with HBS-EP buffer in a flow rate of 5 µL/min. Biotinylated α-D-mannose-PAA with the same concentration was immobilized as reference on flow cell 1. Binding analyses were carried out with running buffer (HEPES 10 mM, NaCl 150 mM, $CaCl_2$ 10 mM, pH 7.4.) at a flow rate of 20 µl/min. The PA-IL binding of the glycooligomers **AZO-Gal(1,3)-3**, **AZO-Gal(1,3,5)-5**, **EDS-Gal(1,3)-3**, **EDS-Gal(1,3,5)-5** and **AZO-Man(1,3,5)-5** (as control) was examined. 1 µM Pa-IL was incubated with the specific substance at final concentrations of 400 µM, 100 µM, 50 µM, 25 µM, 10 µM, 1 µM and 0.1 µM, in running buffer for 60 minutes at room temperature. 45 µL of each sample were injected over both lanes whereas the binding signal on the reference cell (α-D-mannose-PAA) was subtracted from the β-D-galactose-PAA flow cell during the binding measurement. Each binding cycle consisted of an injection phase for 80 s followed by a 100 s dissociation phase. The chip was regenerated after each run with 100 mM D-galactose in running buffer. The response values were calculated by subtraction of the report point at the beginning of the sample injections (0 s) from the report point at the end of the dissociation phase (210 s). The binding signal obtained by the 1 µM Pa-IL solution in running buffer without inhibitor was set to 100 % binding. This sample was measured 2 times before and 2 times after each measuring cycle. The binding signals of the specific inhibitor was referred to Pa-IL and calculated for relative PA-Il binding in % of PA-IL. Each data point (concentration) represents the mean value (± SEM) of two measurements. The obtained data points for each concentration were plotted with Origin 8.5G and fit with Hill1 equation, allowing for the determination of IC_{50} values (the concentration of inhibitor that results from 50 % binding of PA-IL binding to β-D-galactose-PAA on the sensor chip).

The characteristic sigmoidal shape (see Figure S4) was fitted with the Hill equation to obtain the IC_{50} values *k*:

$$y = Start + (End - Start)\frac{x^n}{k^n + x^n} \qquad (1)$$

where *Start* is the value at 100% binding and equals 100, *End* is the value where no binding is observed anymore and equals 0, *x* corresponds to the concentration and *n* describes the cooperativity [4]. IC_{50} values describe the inhibitory potential of a ligand for a specific receptor which is no direct measure for the affinity of a ligand but can be related to the affinity of the ligand in a competitive experiment e.g. as we have performed with PA-IL and against β-D-galactose.

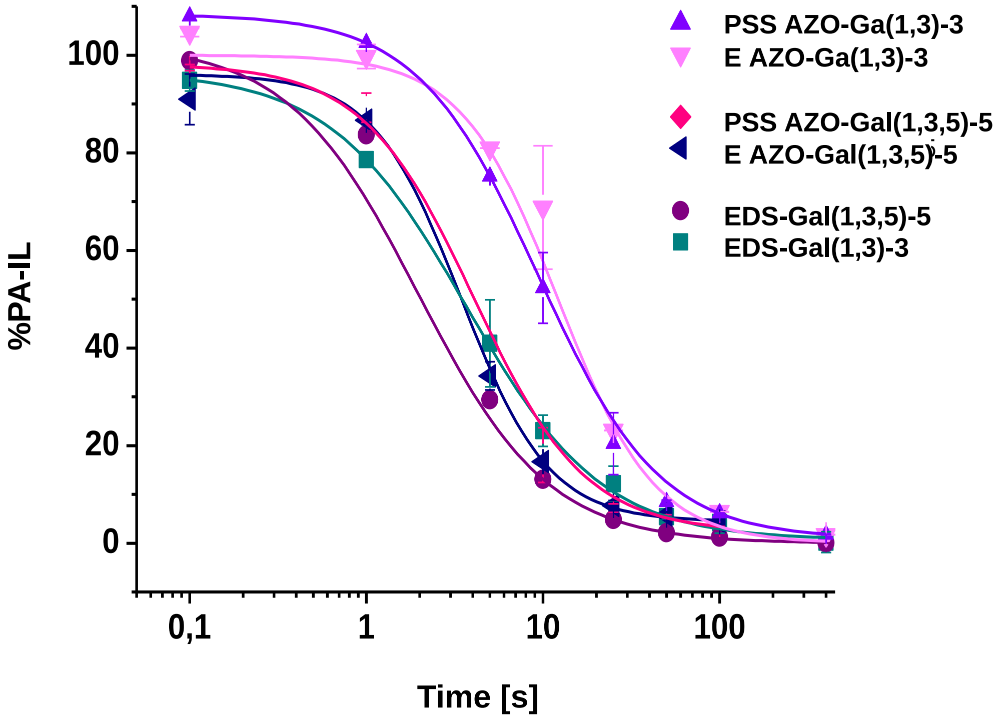

Figure S4: Inhibition curves of AZO-Gal-scaffolds in E/PSS and the EDS-Gal scaffolds as comparison determined by inhibition SPR measurements.

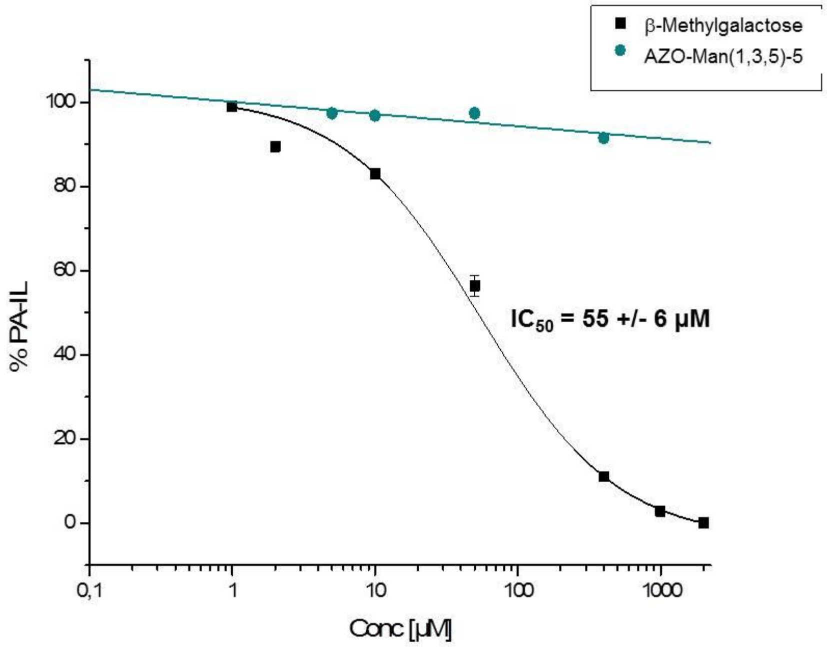

Figure S5: Inhibition curves of controls, **Azo-Man(1,3,5)-5** and β-methyl galactose.

SPR PA-IL direct binding studies

For direct binding studies, glycooligomers *E*-**Azo-Gal(1,3)-3**, *E*-**Azo-Gal(1,3,5)-5**, **Azo-Gal(1,3)-3**-PSS and **Azo-Gal(1,3,5)-5**-PSS were immobilized on Biacore CM5 sensor chips. Two PSS were additionally generated by irradiation of the corresponding *E* structure immobilized on the Biacore sensor chip with light at 360 nm (see irradiation section above for details). Immobilizations were performed by flowing a solution of 2 mM glycoligand for 5 minutes with 10 µL per minute over one flow cell, after activation of it with 0.4 M EDS in water and 0.1 M NHS in water. Remaining active ester groups were capped by injection of 1 M ethanolamine. HBS-P (0.01 M HEPES pH 7.4, 0.15 M NaCl, 0.005% v/v Surfactant P20 in water) was used as running buffer. A second flow cell was immobilized as blank reference cell, by flowing 1 M ethanolamine over the cell after activation with 0.4 M EDS in water and 0.1 M NHS in water. Serial dilutions of PA-IL (25 µM, 10 µM, 5 µM, 1 µM, 0.1 µM and 0 µM) were then flown over the sensor chip at 25 °C with Hepes running buffer (HEPES 10 mM, NaCl 150 mM, $CaCl_2$ 10 mM, pH 7.4.). Binding of lectin was evaluated with Biacore T100 evaluation software, determining K_D affinity constants by employing a steady state affinity model.

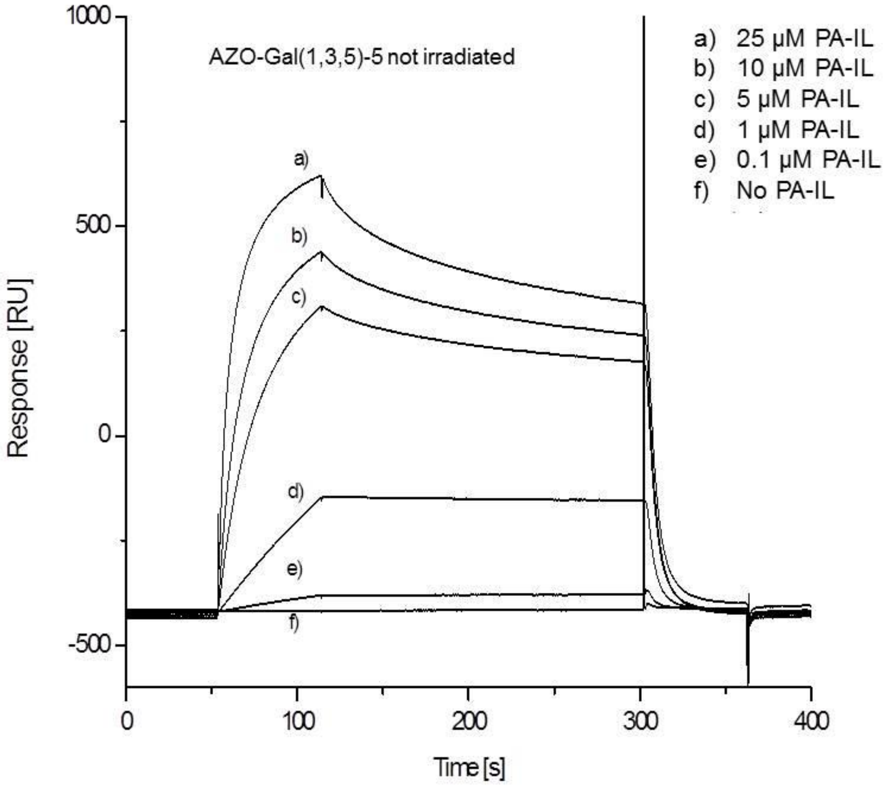

Figure S6: Obtained sensorgram by flowing serial dilutions of PA-IL over the sensor surface functionalized with **Azo-Gal(1,3,5)-5** not irradiated.

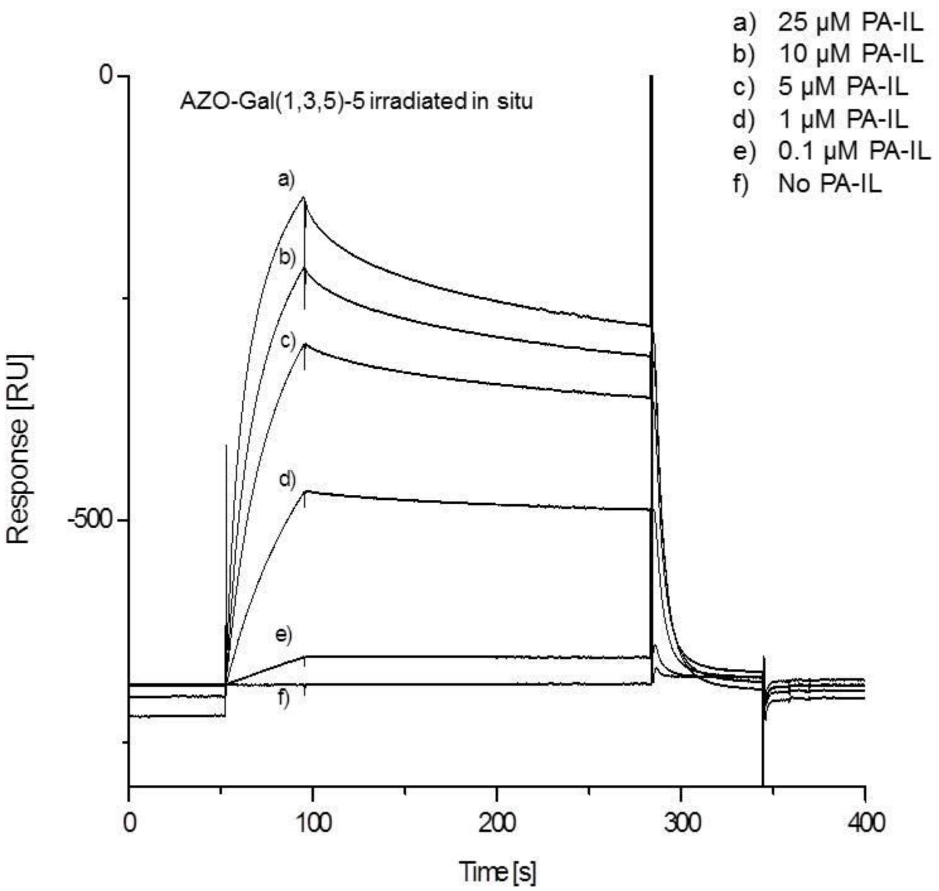

Figure S7: Obtained sensorgram by flowing serial dilutions of PA-IL over the sensor surface functionalized with **Azo-Gal(1,3,5)-5** irradiated in situ.

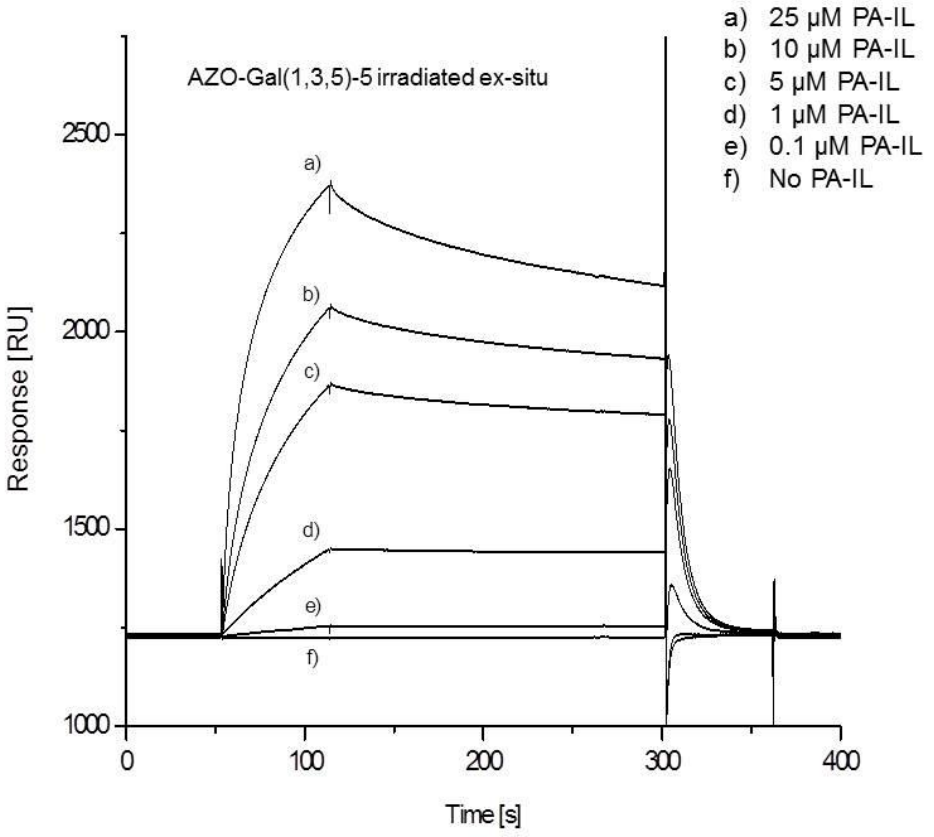

Figure S8: Obtained sensorgram by flowing serial dilutions of PA-IL over the sensor surface functionalized with **Azo-Gal(1,3,5)-5** irradiated ex situ.

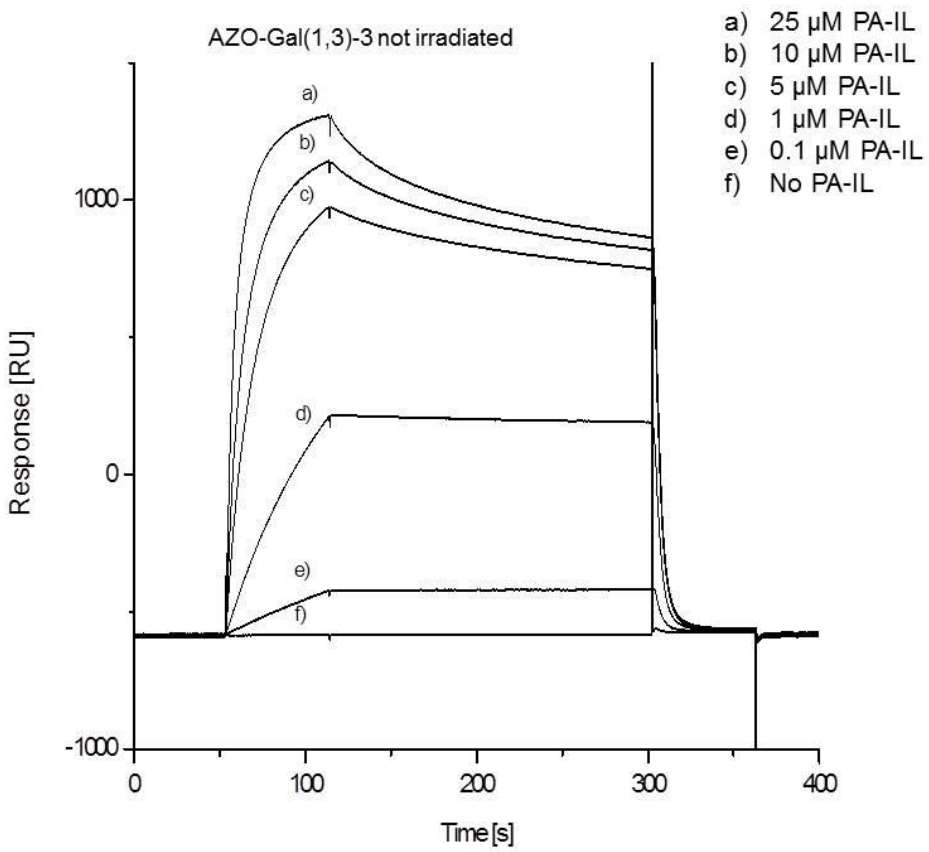

Figure S9: Obtained sensorgram by flowing serial dilutions of PA-IL over the sensor surface functionalized with **Azo-Gal(1,3)-3** not irradiated.

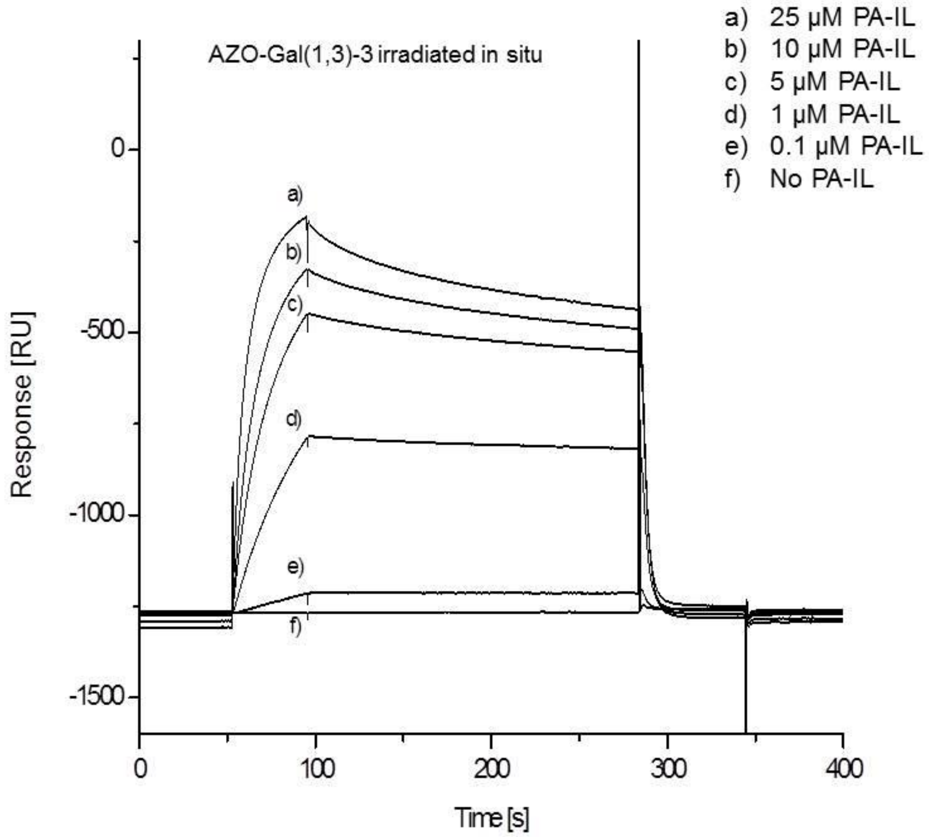

Figure S10: Obtained sensorgram by flowing serial dilutions of PA-IL over the sensor surface functionalized with **Azo-Gal(1,3)-3** irradiated in situ.

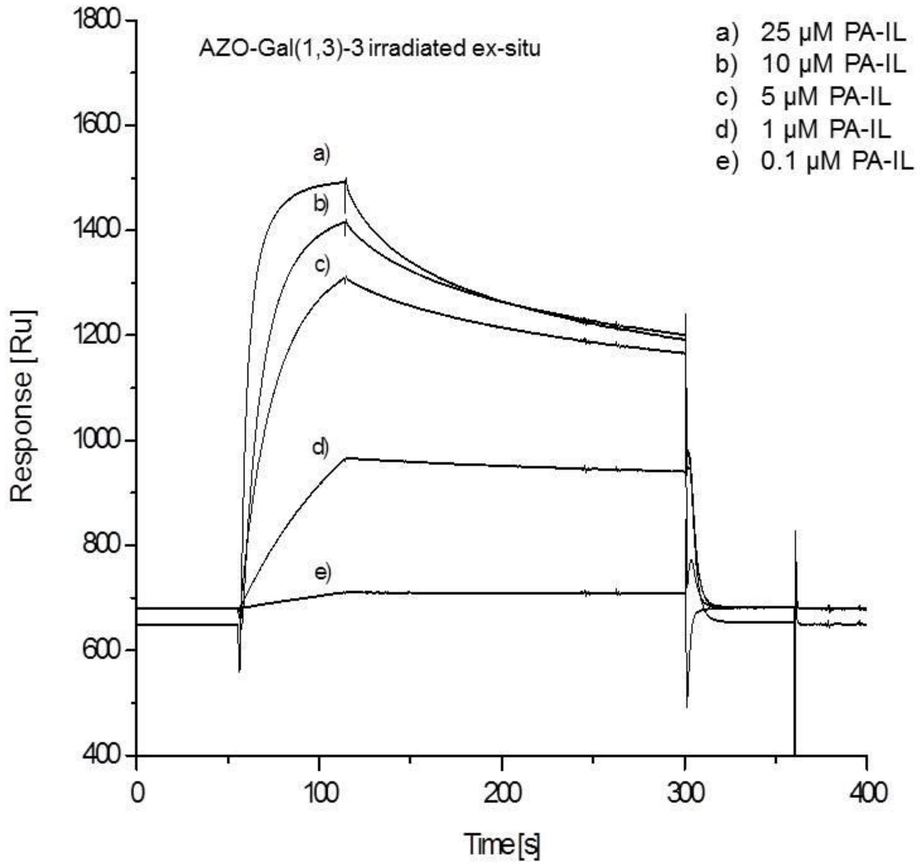

Figure S11: Obtained sensorgram by flowing serial dilutions of PA-IL over the sensor surface functionalized with **Azo-Gal(1,3)-3** irradiated ex situ.

Computational modelling

Computational modelling of **Azo-Gal(1,3)-3** and **Azo-Gal(1,3,5)-5**

The structural models of Azo-Gal(1,3)-3 and Azo-Gal(1,3,5)-5 in *E*- and in all-*Z*-configurations of the connecting azobenzene group have been represented in a crystal structure of Pseudomonas aeruginosa Lectin LecA, complexed with 1-methyl 3-indolyl-β-D-galactopyranoside at 1.45 Å resolution.
The PA-IL protein structure has been inferred from the Protein Data Bank (PDB code 4ljh).
Since this is a very recent and well resolved crystal structure co-crystallized with galactopyranoside analogues, the divalent and the trivalent linker could be modeled to bridge two binding sites at once on the tetrameric protein with a *grafting from* approach:
the galactose ligands were extended by a few atoms belonging to the linker backbone until the rest of the backbone could be matched up and the final structure was then obtained by energy minimization, employing 1,000 steps of steepest descent followed by 10,000 steps of conjugate gradient minimization in implicit solvent, using the Amber12 [5]. Force field parameters were taken from the general Amber force field (GAFF)[6], amended by parameters to adequately represent the Azo-moieties.[7] Additionally, in the supporting information we show that the force field parametrisation is agreeing very well with quantum mechanically derived structures at the B3LYP/6-31G(d,p) level of theory using Gaussian03. [http://www.gaussian.com/g_misc/g03/citation_g03.htm]
The pictures have been rendered using VMD.[8]

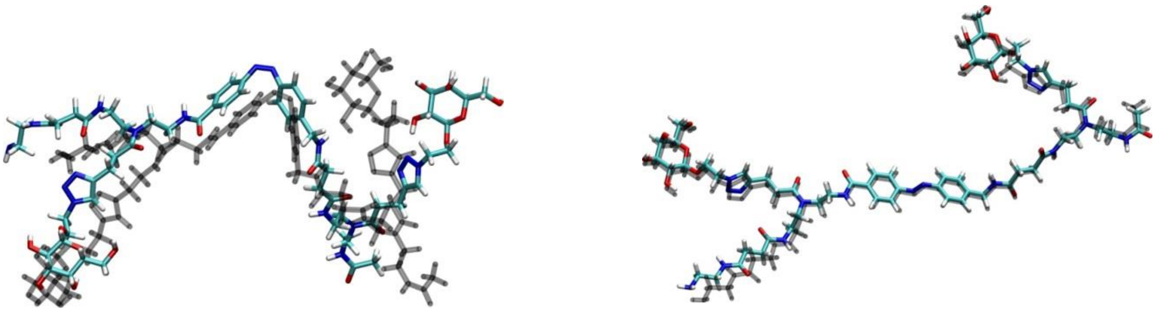

a) b)

Figure S12: **Azo-Gal-(1,3)-3** structures optimized quantum mechanically (QM) at the B3LYP/6-31G(d,p) level of theory (Gaussian03), colored representations; and with molecular mechanics (MM) in the gas phase (AMBER11 with GAFF), gray representation. The structures have been superimposed and aligned as to give a minimal RMSD difference of **a)** 1.317, PSS(*cis*)-state and **b)** 0.230 for the *E*(*trans*)-state. This indicates that close to the overall minimum (with all torsions in their minimum energy (trans-) states) the agreement between MM and QM is easier to achieve, as expected. However, also the gross conformational properties agree reasonably well for the higher energy state represented by structures in **a)**.

Table S1: Selected **Azo-Gal-(1,3,5)-5** showing the versatility of the polymer structure upon UV-irradiation. All configurations shown have been generated from the low energy configuration of the all-*trans* state (azo-moieties and all other bonds) A2 by reorienting the side chains either up (denoted **u** in the chart below) or down (**d**) with respect to the reference structure A2 which has the side chains in an **u-u-u** configuration. We then isomerize the linker from *trans* to *cis* for each azo-group. In the models shown, this involved a rigid rotation around the central N=N bond, followed by a short minimization to relax the structure, leading to the conformations shown in column 1. Note that the molecule is not planar; in A2 and D2, the leftmost galactose residue is approximately pointing in and out of the plane of the paper. Only structures are shown that can potentially bind to PA-IL by bridging two binding sites, other possible conformations such as **u-d-u** have been excluded.

	**1 (all PSS(*cis*)-state)**	**2 (all *E*(*trans*)-state)**
	**u-u-u configuration**	
**A**	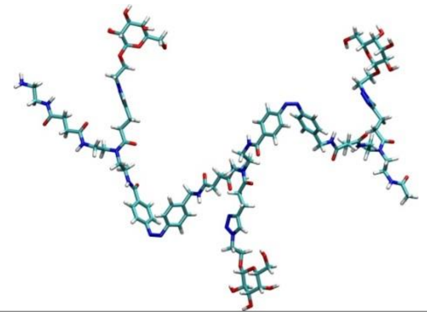	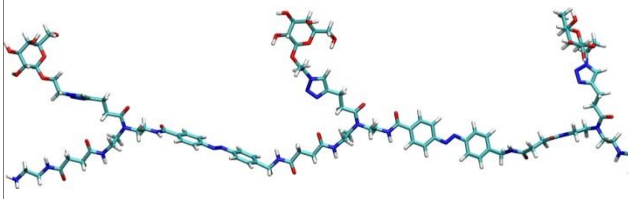
	**u-u-d configuration**	
**B**	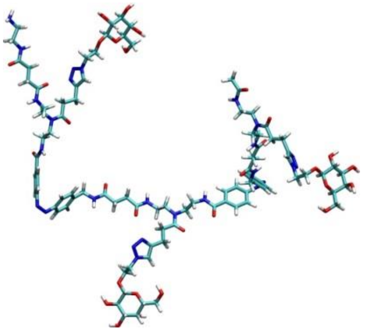	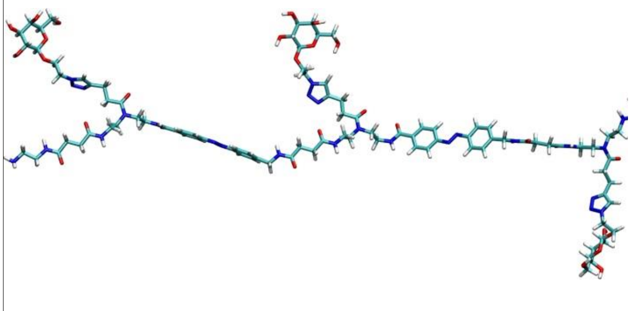

Table S1 continued.

	1 (all PSS(*cis*)-state)	2 (all *E*(*trans*)-state)
	**d-u-u configuration**	
**C**	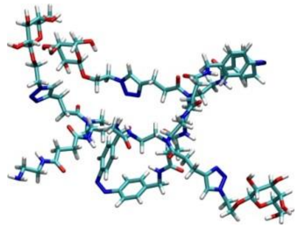	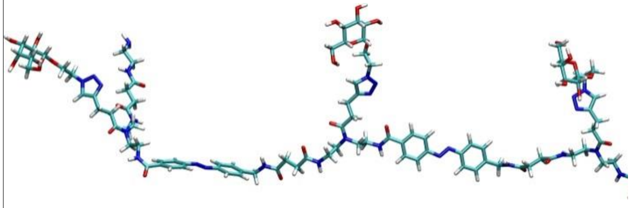
	**d-d-u configuration**	
**D**	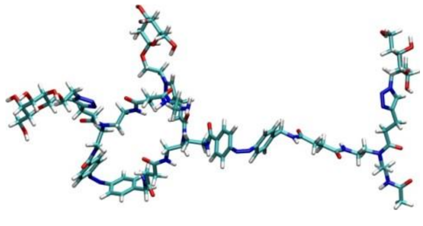	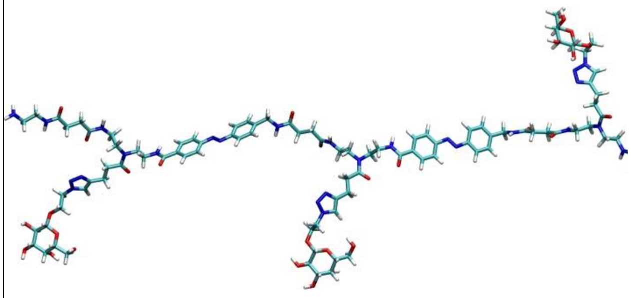

References

[1] Rück-Braun, K., et al. *Synthesis* **2009**, 4256-4267.

[2] Priewisch, B., Rück-Braun, K *J. Org. Chem.* **2005,** *70,* (6) 2350-2352.

[3] Behrendt, R., et al. *J. Pept. Sci.* **1999,** *5,* (11) 519-529.

[4] Prinz, H. *J. Chem. Biol.* **2010**, *3*, 37–44.

[5] Case, D. A.; Darden, T. A.; Cheatham, T. E.; Simmerling III, C. L.; Wang, J.; Duke, R. E.; Luo, R.; Walker, R. C.; Zhang, W.; Merz, K. M., et al. *AMBER11* University of California: San Francisco, **2010**.

[6] Wang, J.; Wolf, R. M.; Caldwell, J. W.; Kollman, P. A.; Case, D. A., *J. Comput. Chem.* **2004,** *25* (9), 1157-1174.

[7] Duchstein, P.; Neiss, C.; Görling, A.; Zahn, D., *J. Mol. Model.* **2012,** *18* (6), 2479-2482.

[8] Humphrey, W.; Dalke, A.; and Schulten, K. VMD-Visual Molecular Dynamics, *J. Mol. Graphics*, **1996,** *14,* 33-38.

Spectral appendix

ESIMS (pos. mode)

Azo-Gal(1,3)-3

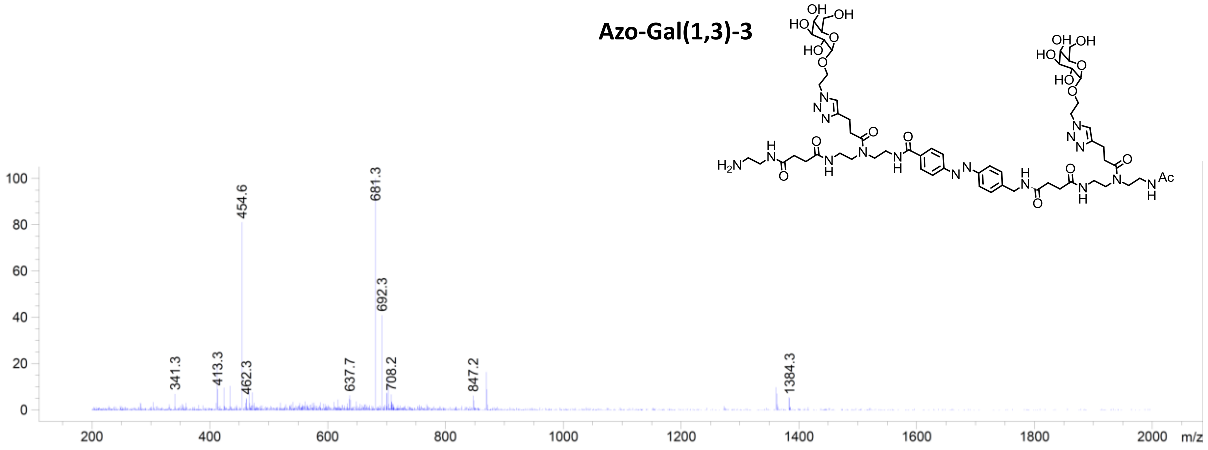

^{1}H NMR

Azo-Gal(1,3)-3

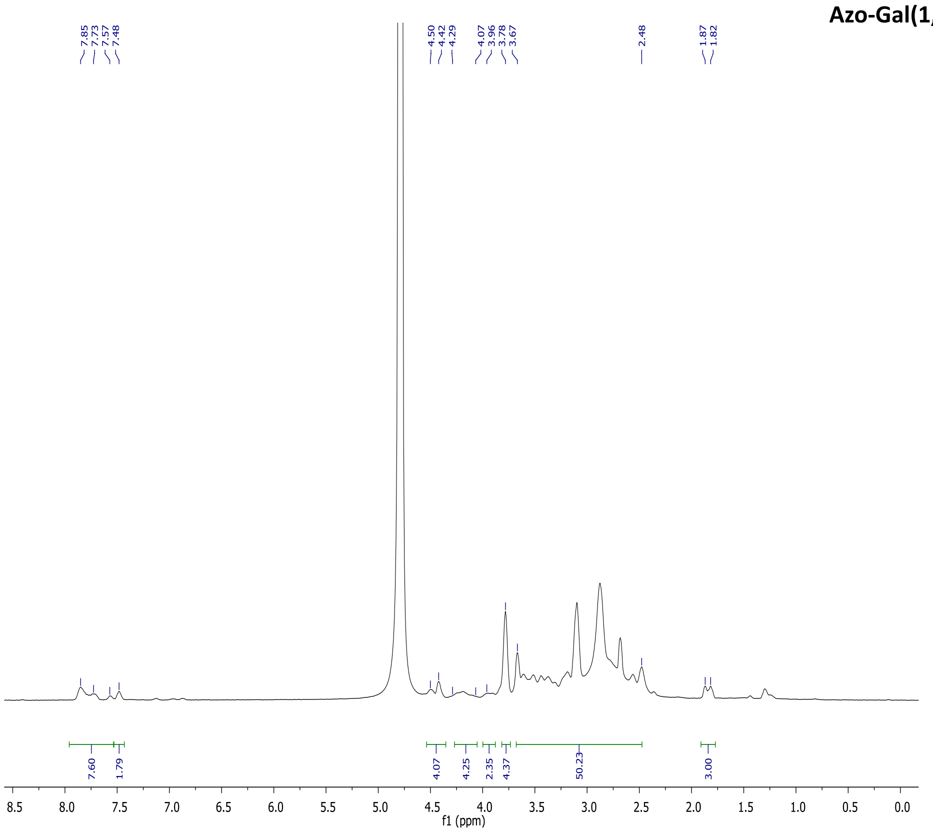

RP-HPLC

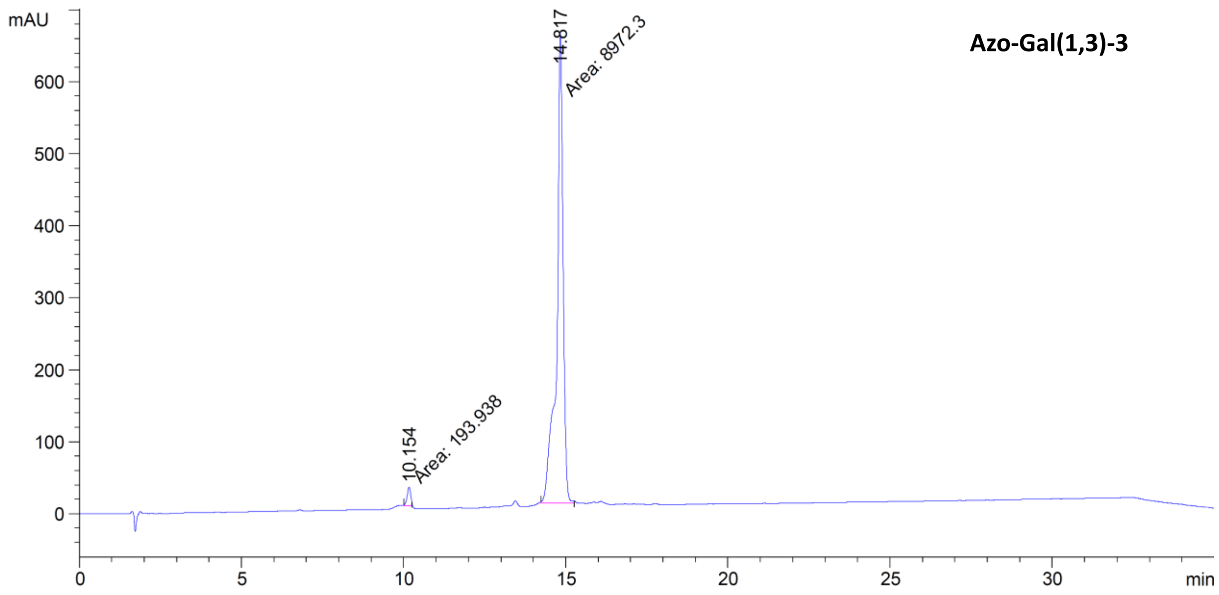

RP-HPLC (5%/95% MeCN/H_2O → 30%/70% MeCN/H_2O in 30 min): t_R = 14.8 min.

ESIMS (pos. mode)

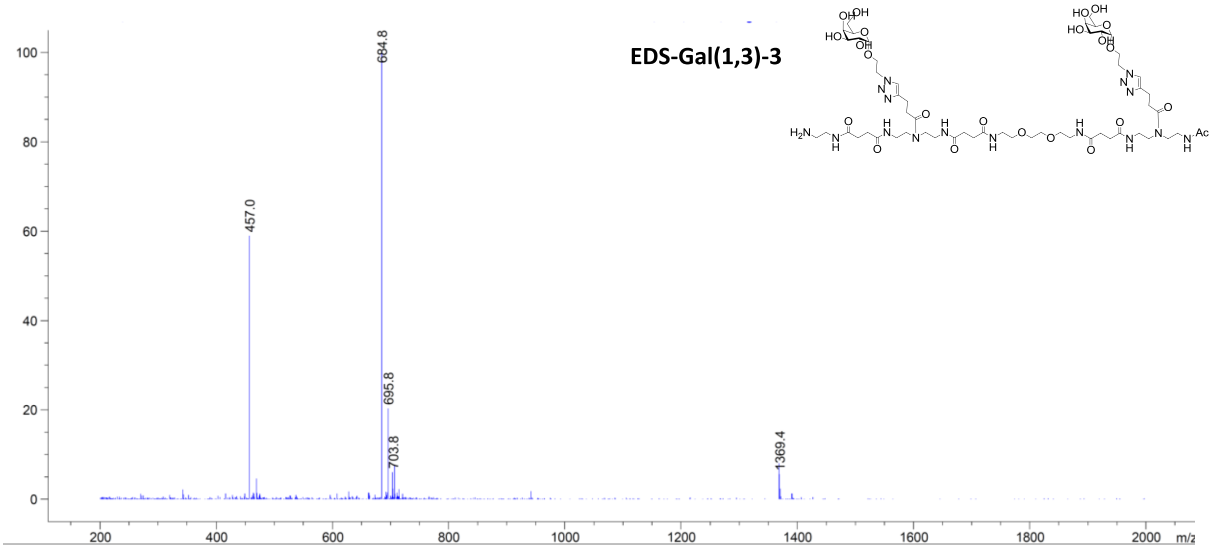

^{1}H NMR

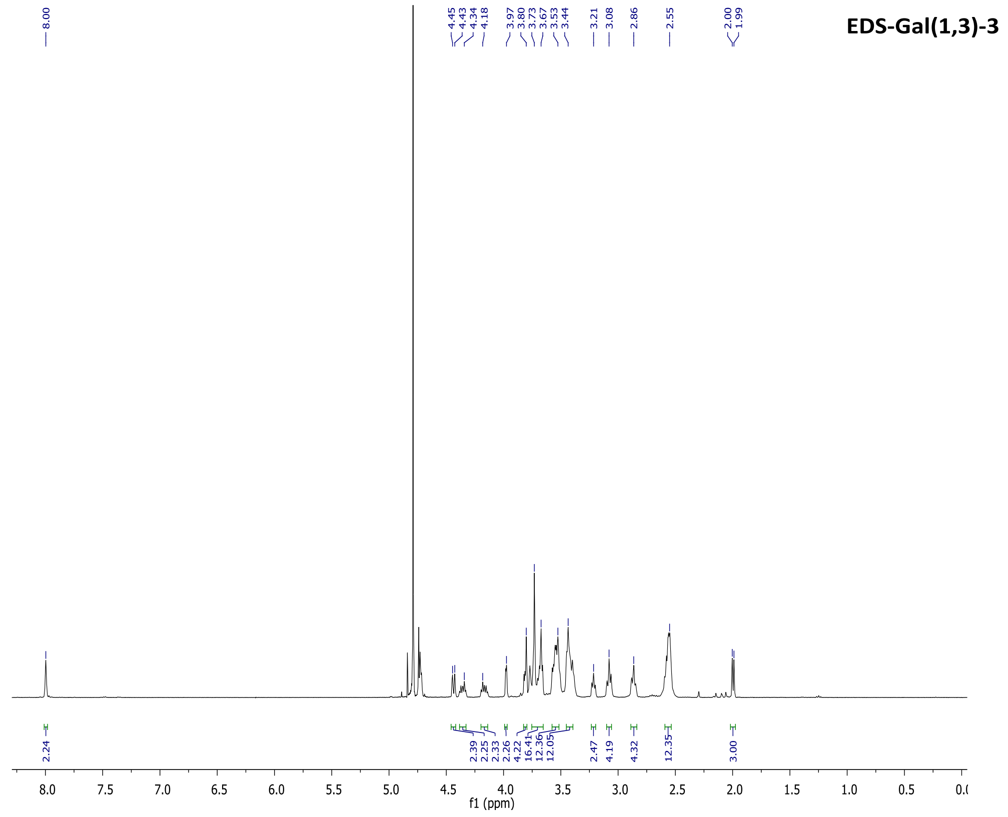

RP-HPLC

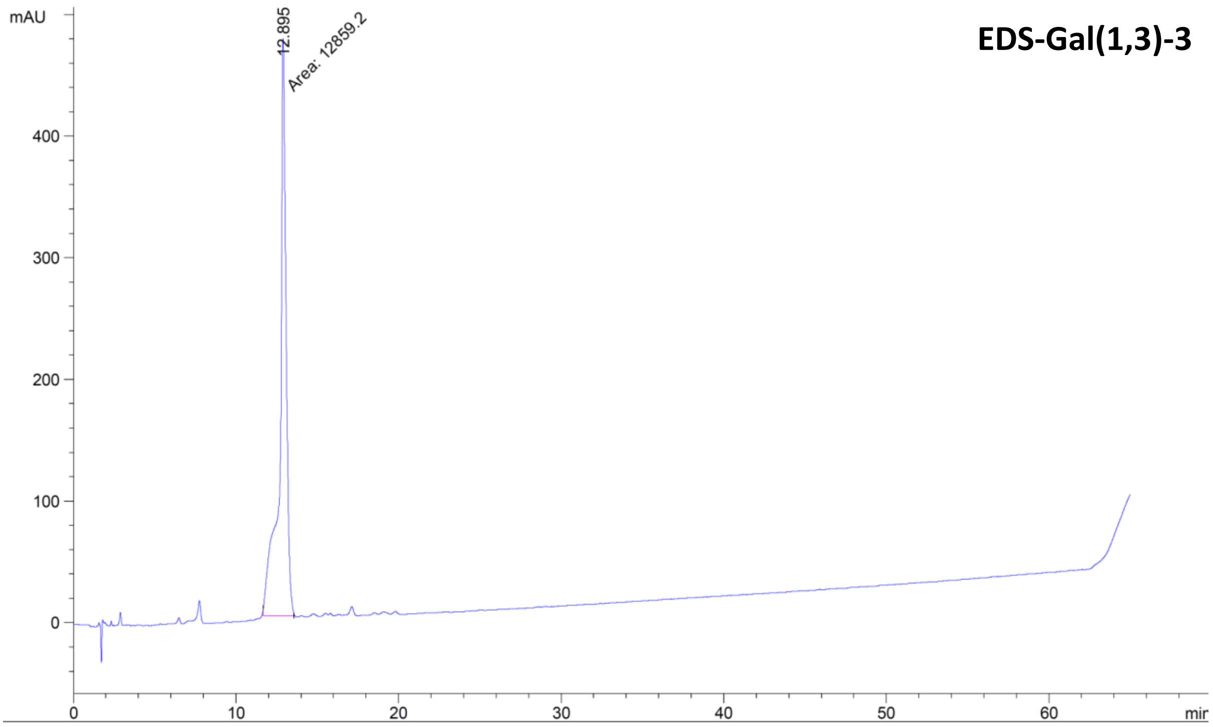

RP-HPLC (5%/95% MeCN/H_2O → 30%/70% MeCN/H_2O in 60 min): t_R = 12.9 min.

ESIMS (pos. mode)

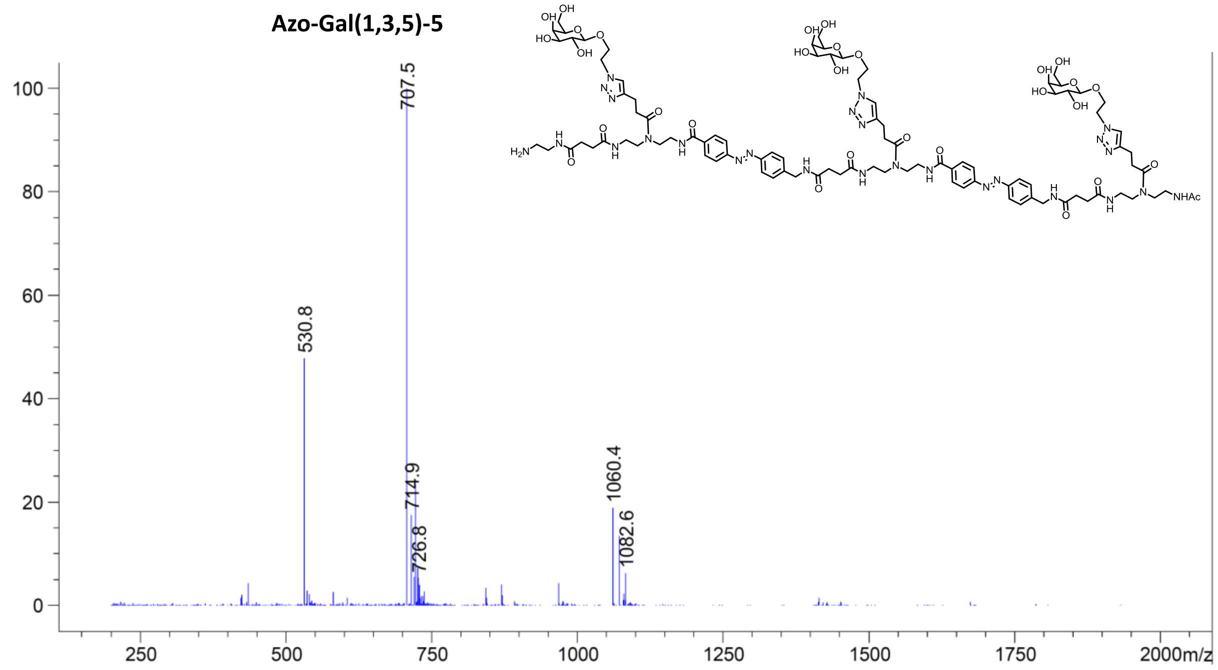

^{1}H NMR

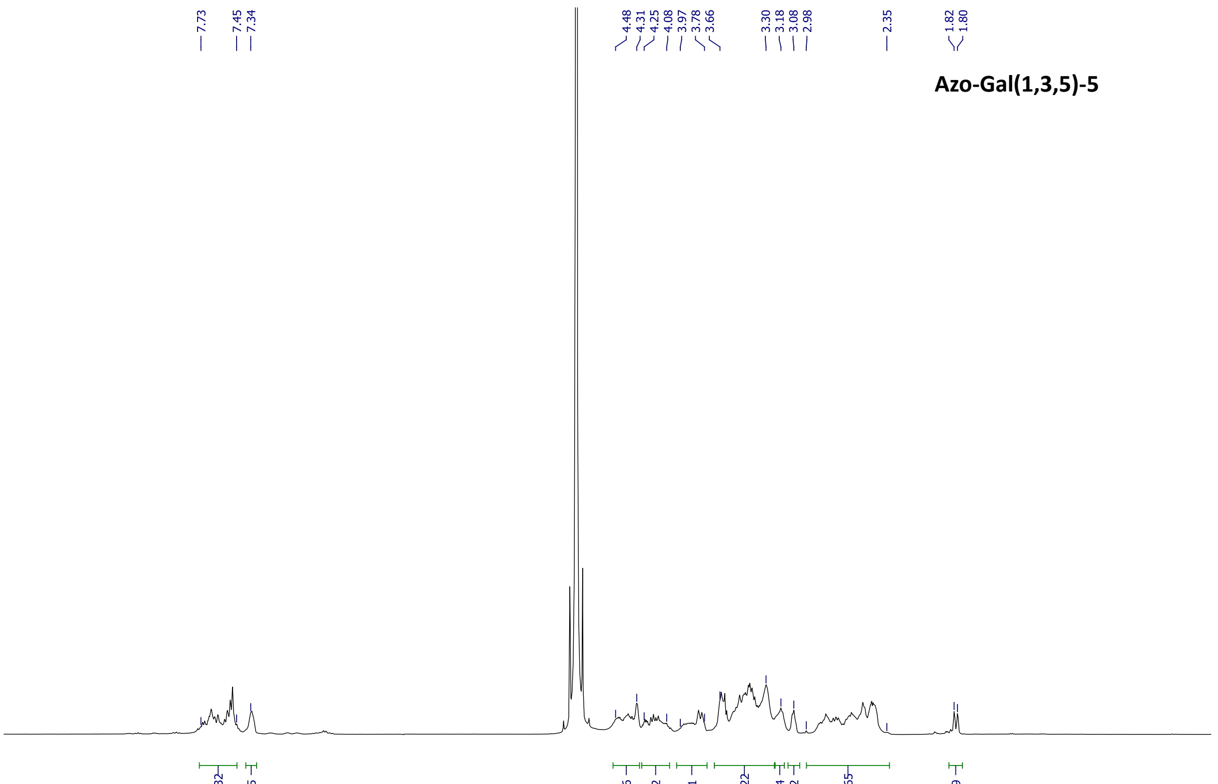

RP-HPLC

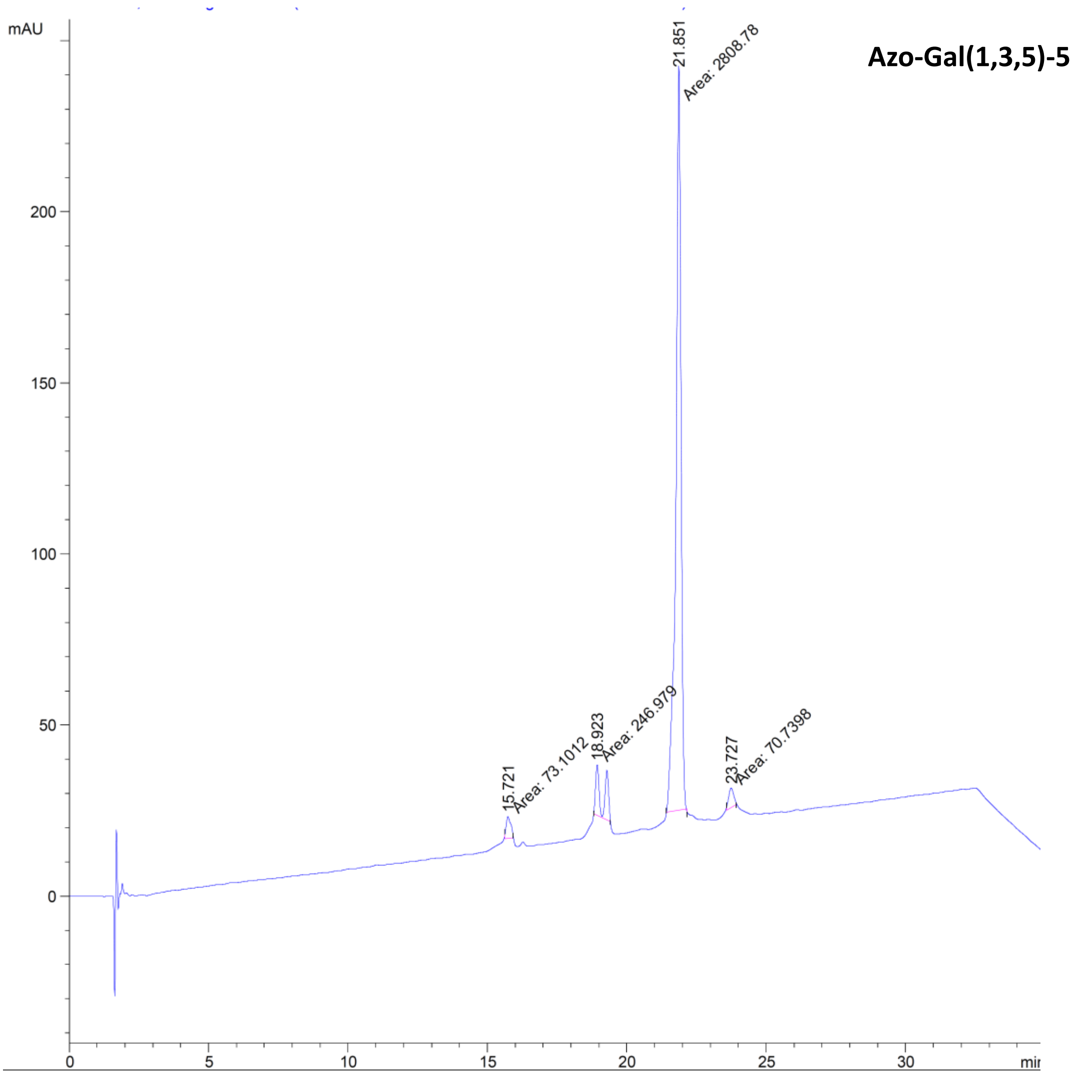

RP-HPLC (5%/95% MeCN/H_2O → 30%/705% MeCN/H_2O in 30 min): t_R = 21.9 min.

ESIMS (pos. mode)

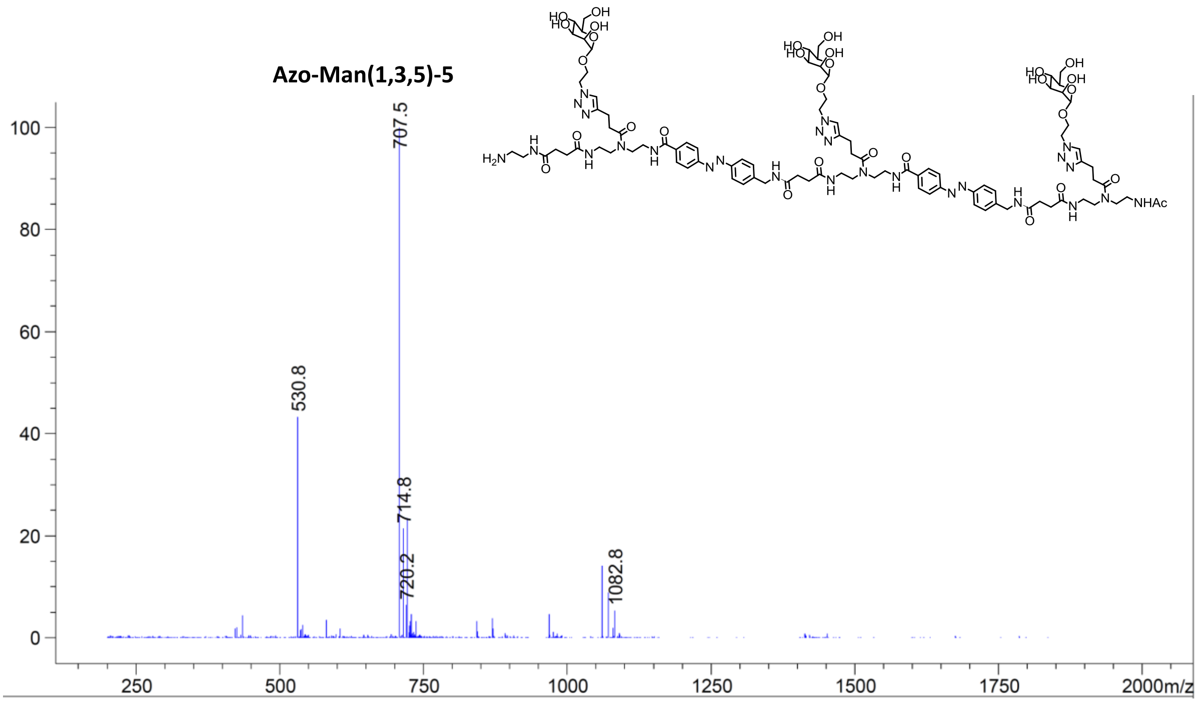

^{1}H NMR

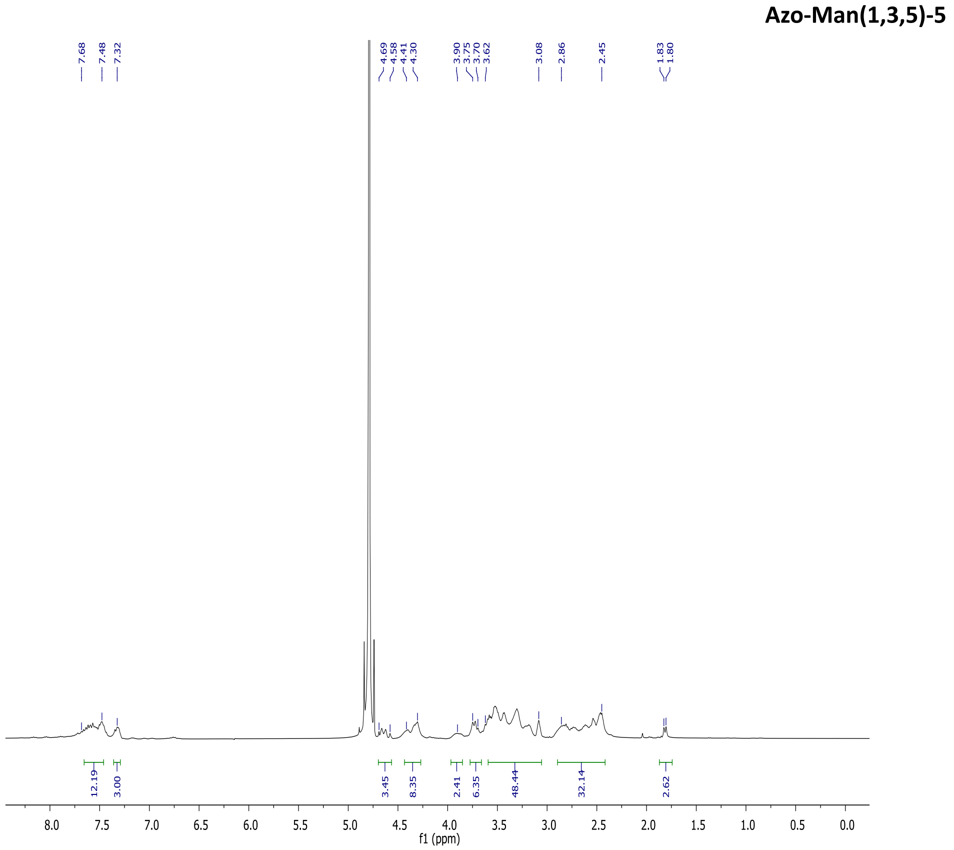

RP-HPLC

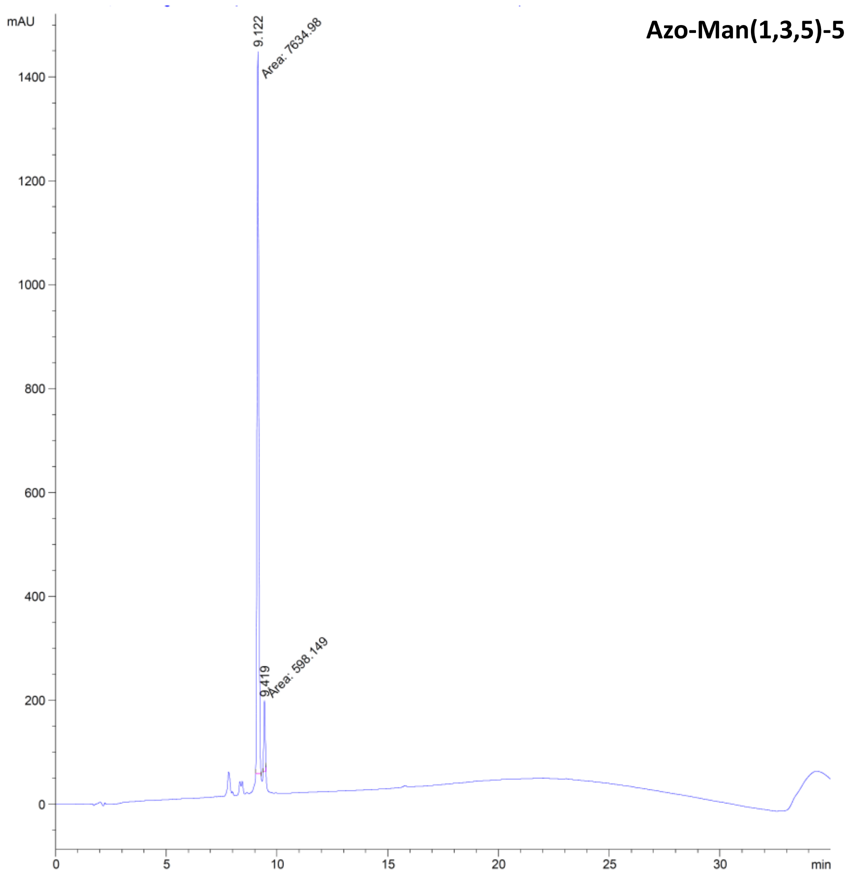

RP-HPLC (5%/95% MeCN/H_2O → 95%/5% MeCN/H_2O in 30 min): t_R = 9.1 min.
